# SubPoE: a subgraph-induced Product-of-Experts framework for spatial multi-omics integration

**DOI:** 10.3389/fgene.2026.1977260

**Published:** 2026-10-22

**Authors:** Yuanfang Li, Wentao Gao, Junhao Ren, Qianran Wei, Xinlei Wang, Liheng Zhang, Ruiting Li, Yadong Wang, Xiao Zhang

**Affiliations:** 1 School of Medicine and Health, Harbin Institute of Technology, Harbin, China; 2 Zhengzhou Advanced Research Institute of Harbin Institute of Technology, Zhengzhou, China; 3 School of Computer Science and Technology, Zhoukou Normal University, Zhoukou, China; 4 School of Artificial Intelligence, Henan University, Zhengzhou, China; 5 College of Animal Science and Technology, Henan University of Animal Husbandry and Economy, Zhengzhou, China

**Keywords:** Product-of-Experts, spatial multi-omics, subgraph-induced learning, SubPoE, uncertainty-aware fusion

## Abstract

**Introduction:**

Spatial multi‐omics technologies jointly profile multiple molecular layers in situ, enabling integrated characterization of tissue organization and cellular states. However, many graph-based approaches remain computationally demanding because they rely on repeated message passing over global graphs, whereas naive local sampling may disrupt cell-level correspondence when multimodal features and graph topologies are not induced synchronously. Moreover, simple concatenation or fixed-weight fusion does not explicitly account for unequal uncertainty between expression‐derived and spatial evidence, making the learned representations sensitive to feature corruption, technical noise and modality inconsistency.

**Methods:**

We present SubPoE, a subgraph-induced Product-of-Experts framework for spatial multi‐omics integration. SubPoE synchronously induces aligned local subgraphs from modality‐specific molecular similarity graphs and a shared spatial graph, preserving molecular‐similarity topology correspondence while reducing per‐iteration graph complexity. Dual‐topology variational graph encoders model expression-derived structure and spatial context as probabilistic experts, which are adaptively fused according to their uncertainty through a Product‐of‐Experts formulation. A composite self-supervised objective further improves robustness and cross‐modal alignment.

**Results:**

Across eight public paired spatial multi‐omics datasets, SubPoE achieves competitive and comparatively stable performance against existing methods. Ablation, sensitivity, robustness and efficiency experiments further support the contributions of synchronous subgraph induction and uncertainty‐aware fusion.

**Discussion:**

These results establish SubPoE as an effective approach for learning topology‐preserving, uncertainty-aware representations of heterogeneous spatial multi‐omics data.

## 1 Introduction

Spatially resolved multi-omics technologies jointly measure multiple molecular layers in their native tissue context (Vandereyken et al., 2023), offering a richer view of tissue architecture, cellular states, and local microenvironments (Moses and Pachter, 2022). By capturing complementary signals from the same cells or spots, they provide more informative inputs than single-modality assays. One key application is spatial domain identification, which groups cells or spots into spatially coherent regions with distinct molecular programs (Rao et al., 2021). In this setting, integrating multiple omics modalities can improve domain delineation, robustness, and biological interpretability.

Graph neural networks have become a central paradigm for the spatial omics integration task (Kipf and Welling, 2017) because they jointly encode molecular profiles and spatial neighborhood structures, allowing information to propagate among nearby cells or spots (Velikovi et al., 2018). STAGATE constructs a spatial neighbor network and uses an adaptive graph attention autoencoder to learn edge-specific neighborhood weights for spatial domain identification (Dong and Zhang, 2022). GraphST integrates gene expression and spatial proximity through graph convolutional representation learning, supplemented by self-supervised contrastive learning to improve the robustness of spatial embeddings (Long et al., 2023). PAST combines a variational graph convolutional autoencoder with prior-informed self-attention to extract spatially structured latent features (L et al., 2023). In contrast, STAMP uses a deep generative topic model to identify interpretable spatial topics and associated gene modules from spatial transcriptomic data (Zhong et al., 2024). Collectively, these methods demonstrate the value of spatially informed representation learning for resolving local tissue organization and spatial domains. However, they are primarily designed for a single transcriptomic modality and do not directly address the integration of heterogeneous molecular measurements from the same spatial locations.

Recent methods have extended graph-based representation learning from spatial transcriptomics to spatial multi-omics integration. SpatialGlue employs modality-specific graph encoders and an attention-based fusion mechanism to learn joint representations from heterogeneous molecular profiles (Long et al., 2024). SpaMV (Liu et al., 2026a) and SSGATE (Lv et al., 2024) use multi-view or multi-branch architectures to capture complementary information across modalities, whereas spaVAE (Tian et al., 2024) adopts generative modeling to characterize shared and modality-specific variation. PRAGA (Huang et al., 2024) further incorporates adaptive graph learning to refine spatial relationships during integration, while CANDIES (Liu et al., 2026b) uses conditional denoising to improve representation robustness. Together, these methods demonstrate the potential of graph-based approaches to integrate complementary molecular signals while preserving spatial context. Despite their different architectures, existing methods generally lack a unified framework for preserving cell-level molecular-similarity topology correspondence and accounting for unequal uncertainty between expression-derived and spatial evidence during fusion. As spatial multi-omics technologies improve in resolution and throughput, they generate larger and denser datasets that are increasingly difficult to analyze efficiently (Chiang et al., 2019). Many existing graph-based methods still rely on repeated message passing over predefined global graphs, which imposes substantial computational and memory costs on large tissue sections (Zeng et al., 2020), while fixed-weight fusion cannot adapt to unequal uncertainty between expression-derived and spatial evidence. Together, these limitations motivate a unified framework that is both computationally efficient and uncertainty-aware.

To address the limitations, we introduce SubPoE, a subgraph-induced Product-of-Experts framework for spatial multi-omics integration. SubPoE synchronously samples cell indices, multi-omics features and their corresponding edges from modality-specific molecular similarity graphs and a shared spatial graph, thereby constructing cell-aligned local views for scalable topology learning. Within each modality, dual-topology variational graph encoders separately model feature-derived structure and spatial context as Gaussian latent experts. Their posterior distributions are combined through Product-of-Experts fusion, allowing the expert with lower uncertainty to contribute more strongly to the modality-specific representation. The fused multi-omics embeddings are then integrated into a joint representation and optimized with a composite self-supervised objective, improving robustness to feature-level perturbations and modality inconsistency.

Across eight paired spatial multi-omics datasets spanning different sequencing platforms and tissue contexts, SubPoE achieved competitive performance against existing methods. Ablation and sensitivity analyses confirmed the roles of synchronous subgraph induction, uncertainty-aware fusion, and cross-modal self-supervision. Cell-level uncertainty analyses, perturbation experiments, and efficiency evaluations further showed that SubPoE adaptively balanced modality-specific evidence, remained robust under controlled feature perturbations and reduced per-step graph-processing cost.

## 2 Methods

### 2.1 Overview of SubPoE

As illustrated in Figure 1, SubPoE is designed around a three-layer architecture: subgraph induction, PoE fusion, and cross-modal self-supervised integration. This design allows spatial multi-omics data to be modeled through expression-derived cellular structure, spatial neighborhood context, and cross-modal consistency. Specifically, SubPoE first preserves cell-level alignment among molecular-similarity topology, spatial topology, and multi-omics features through subgraph-induced dual-topology graph encoding. It then integrates the expression and spatial branches in the latent distribution space through a PoE-based uncertainty-aware fusion module. Finally, a self-supervised objective with stochastic feature masking improves the robustness of the joint representation to local signal loss, technical noise, and modality inconsistency. In the present evaluation, the primary downstream task is spatial domain identification based on clustering of the integrated representation.

**FIGURE 1:**
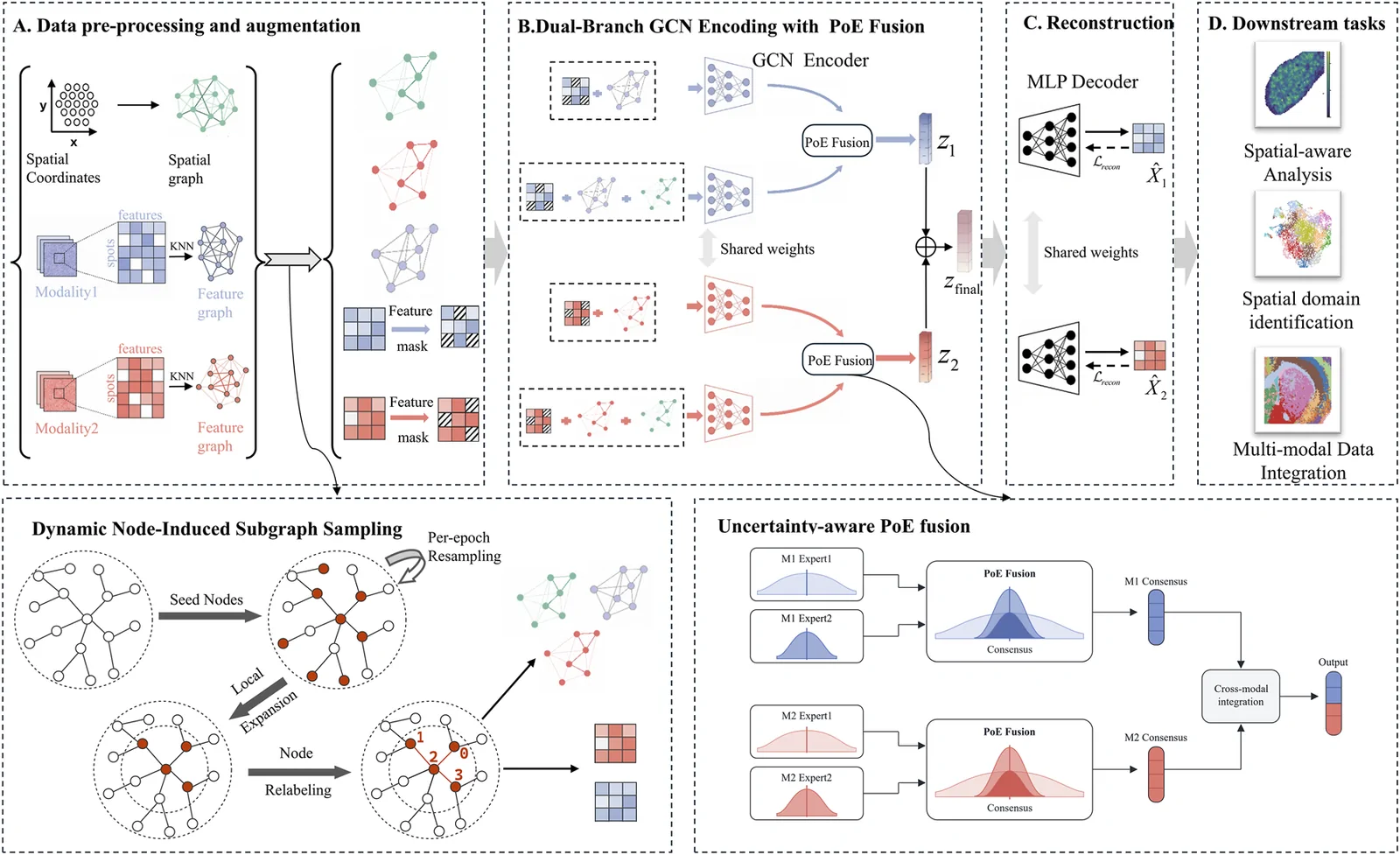
Overall framework of SubPoE **(A)** Given paired spatial multi-omics data and spatial coordinates, SubPoE constructs a shared spatial graph and modality-specific molecular similarity graphs, while random feature masking generates augmented views. Dynamic node-induced subgraphs are sampled through per-epoch seed-node resampling and neighborhood expansion to capture local structures efficiently. **(B)** The original and masked features of each modality are processed by shared-weight dual-branch GCN encoders. An uncertainty-aware PoE module combines the corresponding expert posterior distributions according to posterior precision, producing robust modality-specific embeddings that are subsequently integrated into a unified representation. **(C)** Shared-weight MLP decoders reconstruct the modality-specific inputs, with reconstruction objectives regularizing the learned latent space. **(D)** The integrated representation supports spatially aware analysis, spatial domain identification and multimodal data integration.

### 2.2 Preprocessing of the spatial multi-omics dataset

SubPoE takes spatial multi-omics profiles and the spatial coordinates of each tissue section as input. Throughout this study, we use “spatial observation” to denote the matched measurement unit in each dataset, which may correspond to either a cell or a spatial spot depending on the platform. Let X = { $x_{i}^{\left(m\right)}$ |*i* = 1, 2, …, *N*; *m* = 1, 2, …, *M*} denote the multimodal input, where $x_{i}^{\left(m\right)}$ is the feature vector of cell *i* in the m-th molecular modality, *N* is the number of spatial observations, and *M* is the number of molecular modalities available in the current dataset (Liu et al., 2023). In this study, the available modalities comprise RNA together with ADT or ATAC measurements (Ben-Chetrit et al., 2023). Before modality-specific preprocessing, spatial observations are matched by intersecting their identifiers across all available modality matrices (Jiang et al., 2023). The matrices are then restricted to the shared observation set and arranged in the same order. This alignment ensures that graph construction, local subgraph induction (Zhang et al., 2023), latent fusion and reconstruction operate on corresponding observations across modalities.

For the RNA profiles, highly variable genes are selected to retain informative transcriptomic variation while reducing noise from low-abundance and weakly variable genes. Specifically, the top $N_{HVG}$ highly variable genes are identified from the raw count matrix using the Seurat procedure (Stuart et al., 2019). The resulting expression matrix is normalized for library size, log-transformed to stabilize its distribution and standardized across the selected genes, as shown in Equation 1.

$$X_{ij}^{\text{RNA}}=\frac{\log\left(1+\frac{sC_{ij}^{\text{RNA}}}{\sum_{k}C_{ik}^{\text{RNA}}}\right)-\mu_{j}}{\sigma_{j}+\varepsilon }$$ (1)

where $C_{ij}^{\text{RNA}}$ is the raw count of gene *j* in cell *i*, *s* = 10,000 is the target library size, and $\mu_{j}$ and $\sigma_{j}$ are the mean and standard deviation of the log-normalized expression of gene *j* across cells.

Meanwhile, the ADT and ATAC profiles are processed separately to accommodate their distinct distributional properties. The raw feature matrix is transformed using TF–IDF, followed by row-wise $L_{1}$ normalization and a log (1 + x) transformation, as defined in Equation 2. When LSI is enabled, randomized singular value decomposition is applied to derive a low-dimensional representation. The first LSI component is removed to reduce dominant technical variation, and the remaining components are standardized. Following modality-specific preprocessing, multimodal feature matrices are independently rescaled to [0,1] by column-wise min–max normalization, reducing cross-modal scale differences and improving numerical stability during joint optimization.

$$L_{ij}^{q}=\log\left(1+\frac{C_{ij}^{q}\left[\log\left(\frac{1+N}{1+{\text{df}}_{j}}\right)+1\right]}{\sum_{k=1}^{P_{q}}C_{ik}^{q}\left[\log\left(\frac{1+N}{1+{\text{df}}_{k}}\right)+1\right]}\right),q\in \left\{\text{ADT},\text{ATAC}\right\}$$ (2)

where $C_{ij}^{q}$ is the raw value of feature *j* in cell *i,* N is the number of spatial observations, and ${\text{df}}_{j}$ is the number of cells with nonzero values for feature *j*.

### 2.3 Subgraph-induced dual-topology graph encoding

Full-graph message passing becomes increasingly demanding as the number of spatial observations grows and repeatedly propagates information through all predefined connections, including potentially redundant or low-quality edges. SubPoE therefore treats node-induced subgraphs as consistent local modeling units rather than solely as a computational shortcut. At the global level, a molecular similarity graph is constructed for each omics modality, while a shared spatial graph is derived from the physical coordinates. These graphs contain the same spatial observations but describe complementary molecular and spatial neighborhood relationships.

To make the construction of these global graphs explicit, let $s_{i}$ in $R^{2}$ denote the physical coordinate of observation *i*, and let $x_{i}^{\left(m\right)}$ denote its preprocessed feature vector in the m-th molecular modality, *m* = 1, 2, …, *M*. A shared spatial graph is first constructed by identifying the $k_{\text{spatial}}$ nearest neighbors of each cell according to the Euclidean distance between their physical coordinates, as shown in Equation 3:

$$d_{ij}^{\text{spatial}}={\left\|s_{i}-s_{j}\right\|}_{2}\;N_{i}^{\text{spatial}}={\text{KNN}}_{k_{\text{spatial}}}\left(i;{\left\{d_{ij}^{\text{spatial}}\right\}}_{j\neq i}\right)$$ (3)

The corresponding global spatial edge set is defined as $E_{global}^{spatial}=\left\{\left(i,j\right)\left|\;i,j\in V_{global}\;j\in N_{i}^{spatial},\right.\right\}$ . In parallel, a modality-specific molecular graph is constructed from the preprocessed feature matrix of each molecular modality. The molecular similarity between cells *i* and *j* in modality *m* is measured using the Pearson correlation coefficient, as defined in Equation 4:

$$r_{ij}^{m}=\frac{{\left(x_{i}^{m}-{\bar{x}}_{i}^{m}\right)}^{T}\left(x_{j}^{m}-{\bar{x}}_{j}^{m}\right)}{{\left\|x_{i}^{m}-{\bar{x}}_{i}^{m}\right\|}_{2}{\left\|x_{j}^{m}-{\bar{x}}_{j}^{m}\right\|}_{2}},m=1,2,\ldots ,M$$ (4)

where ${\bar{x}}_{i}^{m}$ is the mean of the entries in $x_{i}^{m}$ .

For each observation, the $k_{m}$ observations with the highest molecular similarity are retained according to Equation 5:

$$N_{i}^{m}=TopK_{\left(k_{m}\right)}\left({\left\{r_{ij}^{m}\right\}}_{j\neq i}\right),E_{global}^{m}=\left\{\left(i,j\right)|i,j\in V_{global},j\in N_{i}^{m}\right\}$$ (5)

Equivalently, the binary adjacency matrix of each molecular or spatial graph is given by $A_{ij}^{\left(m\right)}=\left\{\begin{array}{cc} 1, & \left(i,j\right)\in E_{\text{global}}^{\left(m\right)}\\ 0, & \text{otherwise} \end{array}\right.\;m=1,2,\ldots ,M$ for modality-specific molecular similarity graphs and *m = spatial* for the shared spatial graph.

Thus, $E_{global}^{m}$ encodes modality-specific molecular similarity, whereas $E_{global}^{spatial}$ encodes physical proximity shared across modalities. These global edge sets serve as fixed candidate topologies from which aligned node-induced subgraphs are extracted during training.

At each training iteration, SubPoE constructs a local node set through seed-node resampling followed by neighborhood expansion, as shown in Equation 6:

$$V_{s}=\left(V_{\text{global}},V_{sub}\right),s.t.\;\left|V_{s}\right|=V_{sub}$$ (6)

where $V_{\text{global}}$ denotes the complete observation set, $V_{sub}$ denotes the seed-node resampling and neighborhood-expansion procedure P , and $V_{s}$ denotes the active local node set used in the current iteration. Specifically, seed nodes are first resampled at each epoch, and their neighboring nodes are then included through the precomputed neighborhood graph to form the active local node set.

Once the expanded active node set has been obtained, the corresponding edges are synchronously induced from every global molecular similarity graph and the shared spatial graph, as defined in Equation 7:

$$E_{m,raw}^{s}=\left\{\left(u,v\right)\in E_{\text{global}}^{m}|u\in V_{s}\wedge v\in V_{s}\right\}$$ (7)

where $E_{\text{global}}^{m}$ is the global edge set for graph m, *u* and *v* are its endpoint nodes, and $E_{m,raw}^{s}$ contains the edges whose endpoints both lie in $V_{s}$ . This operation retains within-subset relationships while removing connections extending beyond the active node set.

Because the retained nodes inherit their global indices, the local identifiers may be sparse and discontinuous. They are therefore mapped to continuous indices before graph convolution, as shown in Equation 8:

$$E_{m}^{s}=\left\{\left(\phi \left(u\right),\phi \left(v\right)\right)|\left(u,v\right)\in E_{m,\text{raw}}^{s}\right\}$$ (8)

where $\phi \left(\;\right)$ maps a global node identifier to its relative index in the current local subgraph, and $E_{m}^{s}$ is the reindexed edge set for graph *m*. This mapping places all modality-specific and shared spatial graph in the same local coordinate system.

Using the same active node set, the local feature matrix of each modality is extracted from its corresponding full feature matrix as shown in Equation 9:

$$X_{s}^{m}=X^{m}\left[V_{s},:\right],m=1,2,\ldots ,M$$ (9)

where $X^{m}$ and $X_{s}^{m}$ are the full and local feature matrices of modality m, respectively. The shared node set $V_{s}$ preserves cell-level correspondence among multimodal features and their induced graph edges.

Each modality is subsequently encoded through two complementary topology branches, as defined in Equation 10:

$$E_{Branch1}^{m}=E_{s}^{m},E_{Branch2}^{m}=E_{s}^{m}\cup E_{s}^{spatial},m=1,2,\ldots ,M$$ (10)

where $E_{s}^{m}$ is the induced molecular edge set for modality m, $E_{s}^{spatial}$ is the induced spatial edge set, and $E_{Branch1}^{m}$ and $E_{Branch2}^{m}$ define the intrinsic-topology and spatially informed branches, respectively. The first branch captures molecular similarity, whereas the second incorporates spatial neighborhood information into the modality-specific topology.

### 2.4 Product-of-Experts-based uncertainty-aware latent fusion

The aligned local features and induced graph topologies are passed to a dual-branch variational graph encoder (Kingma and Welling, 2014). For each molecular modality *m*, the intrinsic-topology branch aggregates information over its molecular similarity graph, whereas the spatially informed branch operates on the topology augmented with spatial connections. Rather than directly concatenating or averaging their outputs, SubPoE represents the two branches as probabilistic experts (Hinton, 2002).

The Gaussian posterior parameters inferred by the two branches of modality m are given by Equations 11 , 12

$$\mu_{1}^{m},\log\left({\left(\sigma_{1}^{m}\right)}^{2}\right)=GCNEncoder_{1}\left({\tilde{X}}^{m},E_{Branch1}^{m}\right)$$ (11)

$$\mu_{2}^{m},\log\left({\left(\sigma_{2}^{m}\right)}^{2}\right)=GCNEncoder_{2}\left({\tilde{X}}^{m},E_{Branch2}^{m}\right)$$ (12)

where $GCNEncoder_{1}$ and $GCNEncoder_{2}$ denote the two variational graph encoders, $\mu_{1}^{m}$ and $\mu_{2}^{m}$ are the posterior means, and ${\left(\sigma_{1}^{m}\right)}^{2}$ and ${\left(\sigma_{2}^{m}\right)}^{2}$ are the corresponding posterior variances of modality m. The resulting fused posterior is computed according to Equation 13.

$$\mu_{fused}^{m}=\left(\frac{\mu_{1}^{m}}{{\left(\sigma_{1}^{m}\right)}^{2}}+\frac{\mu_{2}^{m}}{{\left(\sigma_{2}^{m}\right)}^{2}}\right){\left(\sigma_{fused}^{m}\right)}^{2},{\left(\sigma_{fused}^{m}\right)}^{-2}={\left(\sigma_{1}^{m}\right)}^{-2}+{\left(\sigma_{2}^{m}\right)}^{-2}$$ (13)

where $\mu_{1}^{m}$ and $\mu_{2}^{m}$ are the expert means, ${\left(\sigma_{1}^{m}\right)}^{2}$ and ${\left(\sigma_{2}^{m}\right)}^{2}$ are their posterior variances, and $\mu_{fused}^{m}$ and ${\left(\sigma_{fused}^{m}\right)}^{2}$ are the parameters of the fused Gaussian posterior. Because posterior precision is the inverse of variance, the less uncertain expert contributes more strongly to the fused representation.

Sampling from the fused posterior produces the modality-specific embeddings, as shown in Equation 14:

$$z_{fused}^{m}∼N\left(\mu_{fused}^{m},{\left(\sigma_{fused}^{m}\right)}^{2}I\right),m=1,2,\ldots ,M$$ (14)

where $z_{fused}^{m}$ is the fused representation of modality *m* and *I* is the identity matrix.

### 2.5 Composite self-supervised objective

SubPoE is optimized through a composite self-supervised objective that links input perturbation, reconstruction-based supervision and latent regularization. Specifically, stochastic feature masking generates perturbed views with preserved cell-level correspondence, self- and cross-modal reconstruction constrains the embeddings to retain modality-specific and shared information, and the final joint objective integrates these signals with variational and latent-consistency regularization for end-to-end training.

#### 2.5.1 Stochastic feature masking for robust representation learning

Stochastic feature masking perturbs the molecular inputs during training, reducing dependence on individual observations and encouraging the encoders to exploit distributed evidence. Each feature entry is independently retained or removed according to a binary mask in Equation 15:

$$M_{ij}∼\text{Bernoulli}\left(1-p_{mask}\right)$$ (15)

where $M_{ij}$ is the mask value for cell *i* and feature *j*, and $p_{mask}$ denotes the masking probability. A value of zero removes the corresponding feature entry, whereas a value of one retains it. The resulting perturbed feature matrix is then obtained as shown in Equation 16.

$${\tilde{X}}^{m}=X_{s}^{m}⊙M^{m}$$ (16)

where $X_{s}^{m}$ is the original feature matrix, $M^{m}$ is the sampled binary mask, and ${\tilde{X}}^{m}$ is the resulting perturbed feature matrix.

Masking is conditionally activated during training to balance feature perturbation against information preservation, as summarized in Equation 17:

$$\left.{\tilde{X}}^{m}=\left\{\begin{array}{c} X_{s}^{m}⊙M^{m},with\;ptobability\;p_{mask}\\ X_{s}^{m},with\;probability\;1-p_{mask} \end{array}\right.\right)$$ (17)

where ${\tilde{X}}^{m}$ is the corresponding input supplied to the encoder after conditional masking. The original and perturbed views retain identical cell indices and graph topologies and are subsequently used for reconstruction and latent-consistency learning.

This perturbation step therefore provides aligned augmented views on which the subsequent reconstruction and latent-consistency constraints operate.

#### 2.5.2 Cross-modal self-supervised reconstruction and latent-consistency constraints

Building on the masked and original views, SubPoE first integrates the modality-specific PoE embeddings into a joint representation for downstream analysis. This integration preserves complementary information across molecular modalities and carries both molecular-topology and spatial-context evidence learned within each modality into a shared latent space. Specifically, the final representation is obtained by concatenating the fused embeddings from available modalities, as shown in Equation 18:

$$z_{final}={∥}_{m=1}^{M}z_{fused}^{m}$$ (18)

where || denotes concatenation along the feature dimension, $z_{fused}^{m}$ is the fused representation of modality m, and $z_{final}$ is the final integrated multi-omics representation.

Shared-weight multilayer perceptron decoders reconstruct the original inputs from the joint embedding according to Equation 19:

$${\hat{X}}^{m}=MLPDecoder_{m}\left(z_{final}\right)$$ (19)

Cross-modal reconstruction provides an additional self-supervised signal by reconstructing each target modality from the fused representation of another available modality, as defined in Equation 20:

$${\hat{X}}_{cross}^{m\leftarrow n}=MLPDecoder_{m}\left(z_{fused}^{n}\right),m,n=1,2,\ldots ,M,m\neq n$$ (20)

where ${\hat{X}}_{cross}^{m\leftarrow n}$ denotes the reconstruction of target modality m from source modality n. Together with stochastic feature masking and self-reconstruction, these paths constrain the model to preserve modality-specific signals and improve cross-modal predictability.

These reconstruction paths define the main observable supervision signals, which are then combined with variational regularization and cell-matched latent alignment in a unified training objective.

#### 2.5.3 Joint optimization objective

Given the masked inputs and reconstruction paths defined above, SubPoE is optimized end to end with a composite objective that jointly enforces input reconstruction, cross-modal predictability, variational regularization and latent-space consistency. The first term is the self-reconstruction loss, which preserves modality-specific information by combining mean squared error with feature-level Pearson correlation distance, as defined in Equation 21:

$$L_{recon}=\sum_{m=1}^{M}\left[MSE\left(X_{s}^{m},{\hat{X}}^{m}\right)+\frac{1-\rho_{Pearson\left(X_{s}^{m},{\hat{X}}^{m}\right)}}{2}\right]$$ (21)

where MSE denotes mean squared error, and $\rho_{Pearson}$ is the Pearson correlation coefficient.

Cross-modal predictability is imposed through a reconstruction loss that penalizes the discrepancy between each target modality and its reconstruction from another modality, as defined in Equation 22:

$$L_{cross}=\sum_{m=1}^{M}\sum_{n=1,n\neq m}^{M}MSE\left(X_{s}^{m},{\hat{X}}_{cross}^{m\leftarrow n}\right)$$ (22)

where each term quantifies the discrepancy between target modality *m* and its reconstruction from source modality *n*.

Variational regularization constrains the branch-specific posterior distributions towards a standard multivariate Gaussian prior, as defined in Equation 23:

$$L_{KL}=\sum_{m=1}^{M}\sum_{b\in \left\{1,2\right\}}D_{KL}\left[N\left(\mu_{b}^{m},{\left(\sigma_{b}^{m}\right)}^{2}\right)∥N\left(0,I\right)\right]$$ (23)

where D_KL (.||.) denotes Kullback-Leibler divergence, *b* indexes the two topology branches, and *I* is the identity matrix.

Cell-matched embeddings from different molecular modalities are further aligned through a combination of mean squared error and cosine distance, as shown in Equation 24:

$$L_{cons,b}=\sum_{m=1}^{M}\sum_{n=m+1}^{M}\left[MSE\left(z_{b}^{m},z_{b}^{n}\right)+1-CosSim\left(z_{b}^{m},z_{b}^{n}\right)\right],b\in \left\{1,2\right\}$$ (24)

where $z_{b}^{m}$ and $z_{b}^{n}$ are the cell-matched latent representations of modalities *m* and *n* produced by branch *b*, and CosSim (.) denotes cosine similarity. Each modality pair is counted once.

The complete optimization objective is given in Equation 25

$$L_{total}=L_{recon}+w_{cross}L_{cross}+\gamma \cdot \left(w_{KL}L_{KL}+w_{z1}L_{cons,z1}+w_{z2}L_{cons,z2}\right)$$ (25)

where $w_{cross}$ , $w_{KL}$ , $w_{z1}$ and $w_{z2}$ control the relative contributions of the corresponding loss terms, while γ is a linear warm-up factor applied during early training to reduce the risk of posterior collapse.

## 3 Results and discussion

### 3.1 Baseline methods and evaluation metrics

To evaluate SubPoE against representative state-of-the-art approaches, we selected nine baseline methods spanning several computational paradigms. The baseline methods were grouped according to their primary architectural characteristics. GraphST and PRAGA were categorized as graph neural network methods. STAGATE, SpatialGlue, and SSGATE represented graph attention-based methods. PAST, STAMP, and spaVAE represented variational generative models, whereas SpaMV represented cross-modal representation learning.

All methods were benchmarked under a unified evaluation protocol to ensure a fair comparison. Spatial transcriptomics methods received only the gene expression matrix and corresponding spatial coordinates, whereas spatial multi-omics methods received the complete paired omics profiles and associated spatial information. For each dataset, all methods were evaluated on the same samples under matched clustering settings, with identical downstream scoring procedures applied to their outputs.

Clustering performance was evaluated using six complementary metrics: normalized mutual information (NMI), adjusted mutual information (AMI), the Fowlkes–Mallows index (FMI) (Fowlkes and Mallows, 1983), the adjusted Rand index (ARI) (Hubert and Arabie, 1985), V-measure and completeness (Compl.). Definitions and mathematical formulations of all metrics are provided in Section 2 of the Supplementary Materials.

Algorithm 1 **SubPoE.**

**Input**: Preprocessed multimodal feature matrices ${\left\{X^{\left(m\right)}\right\}}_{m=1}^{M}$ , spatial coordinates *S*, total epochs *E*, KL warm-up epochs $E_{warm}$ , molecular-neighbor numbers ${\left\{k_{m}\right\}}_{m=1}^{M}$ , spatial-neighbor number $k_{spatial}$ , seed-node number $B_{\text{seed}}$ , subgraph size *B*, masking probability $p_{mask}$ , latent dimension *d*, and loss weights $w_{\text{cross}},w_{\text{KL}},w_{z1},w_{z2}.$

1. Definition: *N* is the number of matched spatial observations; *M* is the number of molecular modalities; *m* ∈ {1,2,*M*} indexes modalities and *b* ∈ {1,2} indexes topology branches.
2. Construct $E_{global}^{spatial}$ from *S* using spatial KNN, and construct $E_{\text{global}}^{m}$ from $X^{m}$ using Pearson-correlation-based KNN for each *m.*
3. Initialize $GCNEncoder_{1}$ , $GCNEncoder_{2}$ , $MLPDecoder_{m}$ , and all trainable parameters Θ.
4. For e = 1,2,E:
5. Resample a seed-node set $V_{seed}\subseteq V_{\text{global}}$ with | $V_{seed}$ | = *min(* $B_{seed}$ *,N).*
6. Starting from $V_{seed}$ , perform neighborhood expansion over the precomputed neighborhood graph until the active node set $V_{s\;}$ reaches *|* $V_{s}$ *| =* *min(* B *,N).*
7. Induce and reindex $E_{s}^{spatial}$ from $E_{global}^{spatial}$ on $V_{s}$ . For each m, slice $X_{s}^{m}$ = $X^{m}\left[V_{s},:\right]$ and induce and reindex $E_{s}^{m}$ from $E_{global}^{m}$ .
8. Set $E_{Branch1}^{m}$ = $E_{s}^{m}$ and $E_{Branch2}^{m}=E_{s}^{m}\cup E_{s}^{spatial}$ .
9. Construct masked views: ${\tilde{X}}^{m}=X_{s}^{m}⊙M_{s}^{m}$ where $M_{s}^{m}∼\text{Bernoulli}\left(1-p_{\text{mask}}\right)$ .
10. For each *b* ∈ {1,2}, compute $\mu_{b}^{m},\log\left({\left(\sigma_{b}^{m}\right)}^{2}\right)={GCNEncoder}_{b}\left({\tilde{X}}^{m},\varepsilon_{Branchb}^{m}\right)$ , and sample $z_{b}^{m}$ by reparameterization.
11. Fuse the two branch posteriors of each modality using PoE
  
$\mu_{fused}^{m}=\left(\frac{\mu_{1}^{m}}{{\left(\sigma_{1}^{m}\right)}^{2}}+\frac{\mu_{2}^{m}}{{\left(\sigma_{2}^{m}\right)}^{2}}\right){\left(\sigma_{fused}^{m}\right)}^{2},{\left(\sigma_{fused}^{m}\right)}^{-2}={\left(\sigma_{1}^{m}\right)}^{-2}+{\left(\sigma_{2}^{m}\right)}^{-2}$

12. Sample $z_{fused}^{m}∼N\left(\mu_{fused}^{m},{\left(\sigma_{fused}^{m}\right)}^{2}I\right),m=1,2,\ldots ,M$ .
13. Construct the joint representation: $z_{final}={∥}_{m=1}^{M}z_{fused}^{m}$

14. For each *m*, reconstruct ${\hat{X}}^{m}=MLPDecoder_{m}\left(z_{final}\right)$ .
15. For each ordered pair (*m*, *n*), reconstruct ${\hat{X}}_{cross}^{m\leftarrow n}=MLPDecoder_{m}\left(z_{fused}^{n}\right),m,n=1,2,\ldots ,M,m\neq n$ .
16. Compute $L_{recon}$ , $L_{cross}$ , $L_{KL}$ , $L_{cons,1}$ , and $L_{cons,2}$ and set *γ =* *min(1, e/* $E_{warm}$ *).*
17. Compute the total objective:
  
$L_{total}=L_{recon}+w_{cross}L_{cross}+\gamma w_{KL}L_{KL}+w_{z1}L_{cons,1}+w_{z2}L_{cons,2}$

18. Update Θ by minimizing $L_{total}$ , and repeat until e = E.
**Output**: Full-data unified representation $z_{final}$ .

### 3.2 Cross-dataset evaluation of spatial domain identification

We benchmarked SubPoE on eight paired spatial multi-omics datasets using a unified evaluation protocol. Performance was assessed with six clustering metrics: normalized mutual information (NMI), adjusted mutual information (AMI), the Fowlkes-Mallows index (FMI), adjusted Rand index (ARI), V-measure and completeness. The detailed quantitative results for all datasets and methods are provided in Supplementary Tables S2–S9.

To assess cross-dataset generalizability, we compared SubPoE with nine competing methods on eight paired spatial multi-omics datasets: Human placenta, Human Lymph Node S1-S3, Human Tonsil S1-S2, and Mouse Brain E13 and E15. As shown in Figure 2a, SubPoE achieved the highest cross-dataset mean for all six metrics, with NMI, AMI, FMI, ARI, V-measure, and completeness scores of 0.41, 0.40, 0.47, 0.31, 0.41, and 0.38, respectively. GraphST was the strongest baseline, with corresponding mean scores of 0.38, 0.37, 0.44, 0.25, 0.38, and 0.36. Relative to GraphST, SubPoE improved the six metrics by 7.07%, 7.24%, 6.32%, 21.17%, 7.07%, and 6.29%, respectively. The metric-wise relative improvements averaged 9.19%, with the largest gain observed for ARI. The consistently highest mean scores indicate that SubPoE achieved the strongest aggregate clustering agreement across different tissues, developmental stages, and modality combinations, rather than deriving its advantage from a single dataset.

**FIGURE 2:**
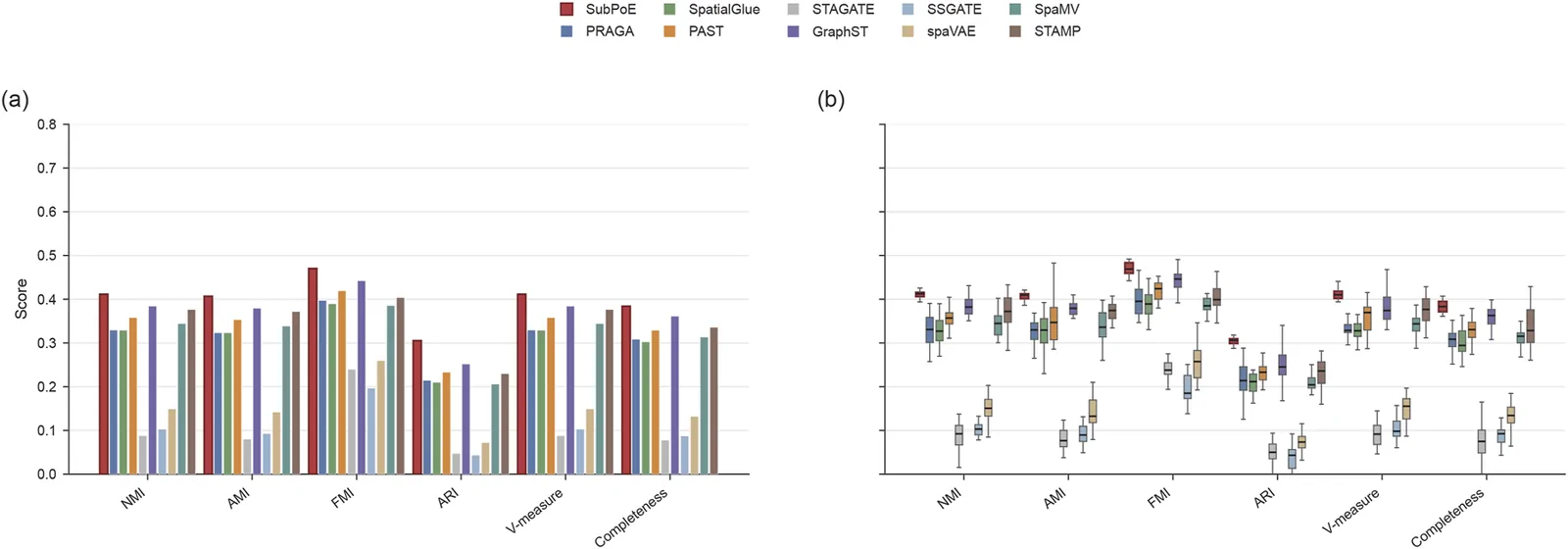
Overall performance and robustness across eight datasets. **(a)** Mean scores across eight datasets. **(b)** Distributions of seed-specific mean scores for six metrics. Figure 3. Spatial clustering patterns and UMAP visualizations generated by different models on the Human Lymph Node S1 dataset.

The seed-specific distributions in Figure 2b further assessed the robustness of each method to training stochasticity. For each method and metric, the score obtained under each random seed was first averaged across the eight datasets, and the resulting seed-specific cross-dataset means were summarized using boxplots. SubPoE maintained high median scores with relatively compact distributions across the six metrics, indicating that its aggregate performance was stable across random initializations. Although the distributions overlapped with those of the strongest baselines for several metrics, SubPoE generally showed a favorable balance between performance and variability. These results support the robustness of its overall cross-dataset performance rather than uniform superiority on every individual dataset or random seed.

### 3.3 Qualitative analysis of spatial domain identification

To assess the multimodal representations learned by SubPoE, we evaluated spatial clustering and low-dimensional embeddings on the Human Lymph Node S1 dataset with paired RNA–ADT measurements. We compared the unimodal RNA and ADT representations, SubPoE, and nine representative baseline methods using the same number of clusters. Spatial maps were used to assess domain continuity and regional organization, whereas UMAP visualizations reflected the compactness and separation of the learned representations.

On the Human Lymph Node S1 dataset, SubPoE recovered broad, spatially coherent compartments across the tissue section (Figure 3). Its spatial assignments formed contiguous regions with distinguishable boundaries, whereas several baselines produced dispersed or locally fragmented patterns. The corresponding UMAP embedding organized the ten clusters into compact and distinguishable regions with limited inter-cluster mixing. By comparison, the original RNA and ADT representations showed greater overlap between neighboring clusters. These observations indicate that SubPoE integrates transcriptomic and protein information into a more structured representation of lymph node heterogeneity.

**FIGURE 3:**
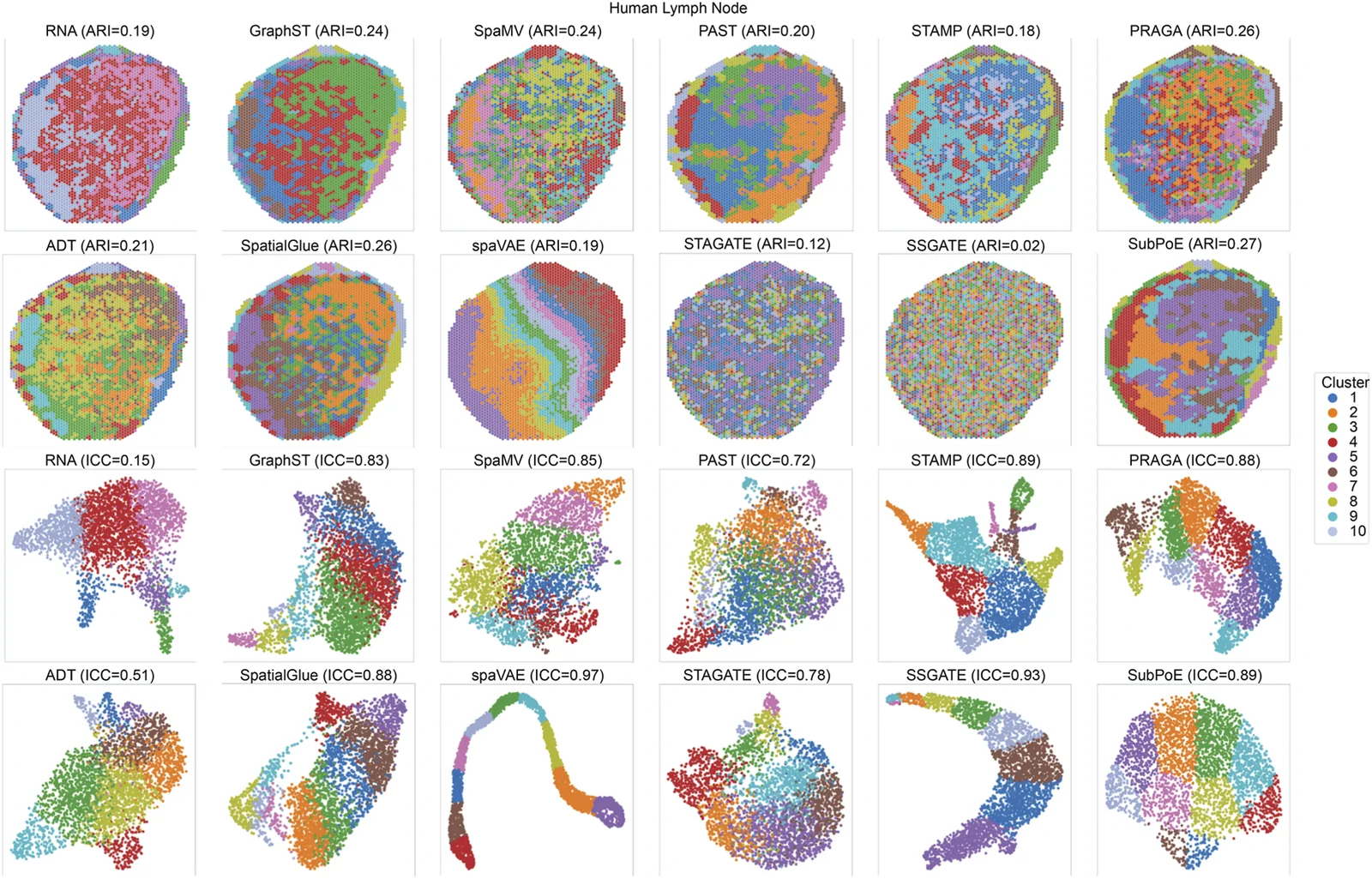
Spatial clustering patterns and UMAP visualizations generated by different.models on the Human Lymph Node S1 dataset.

### 3.4 Ablation and sensitivity analyses

We evaluated the contribution of each SubPoE component by comparing the full model with variants lacking node-induced subgraph sampling, PoE fusion, feature masking, or the Pearson-correlation component of the self-reconstruction loss (Figure 4). On human placenta, the ARI decreased from 0.56 to 0.19 when node-induced subgraph sampling was removed and to 0.22 when PoE fusion was removed. On Human Lymph Node S1, removing node-induced subgraph sampling reduced the ARI from 0.27 to 0.13, whereas removing feature masking reduced it to 0.14. Similar performance decreases were observed on the remaining datasets. Across datasets and metrics, the full model generally performed best, indicating that the four components made complementary contributions to clustering performance.

**FIGURE 4:**
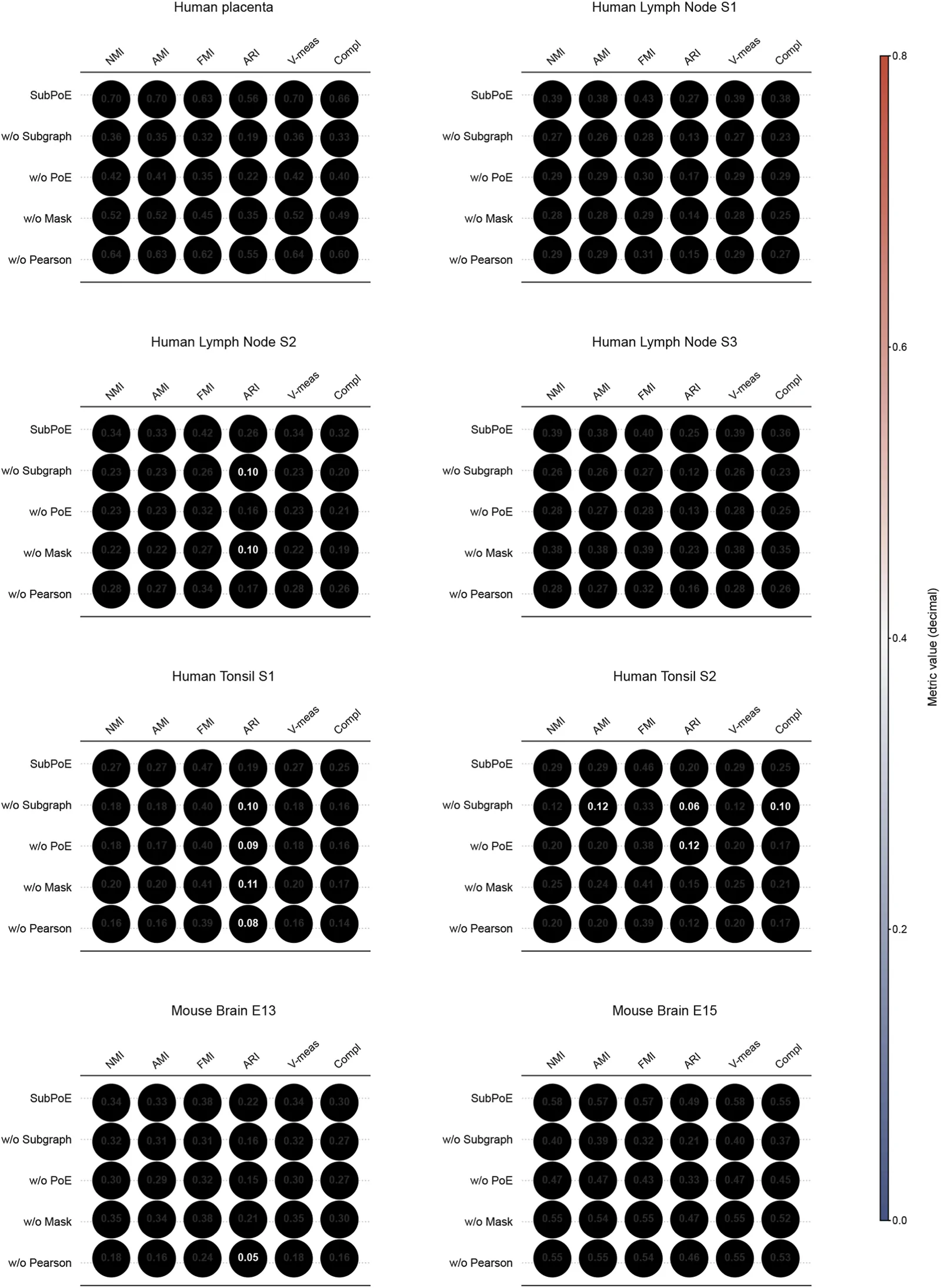
Component ablation analysis of SubPoE across eight spatial multi-omics datasets. Bubble matrices compare the complete model with variants excluding node-induced subgraph sampling, PoE fusion, feature masking, or the Pearson-correlation component of the self-reconstruction loss.

We assessed the sensitivity of SubPoE to eight hyperparameters across eight datasets: subgraph size *s*, masking probability $p_{mask}$ , number of feature neighbors $k_{f}$ , number of spatial neighbors $k_{s}$ , consistency weights λ_1_ and λ_2_, KL-divergence weight *β*, and latent dimension *d* (Figure 5). The subgraph size *s* had the strongest and most dataset-dependent effect on clustering performance. On human placenta, the ARI generally decreased as s increased, whereas Mouse Brain E15 achieved its highest ARI at an intermediate value of *s*. The masking probability p had a smaller effect on most datasets, although human placenta showed a marked decrease after masking was introduced. The feature-neighbor number $k_{f\;}$ and spatial-neighbor number $k_{s}$ produced comparatively modest fluctuations, with no common optimum across datasets. Overall, SubPoE remained stable over broad ranges of most hyperparameters, although the effect and optimal value of s varied across tissues.

**FIGURE 5:**
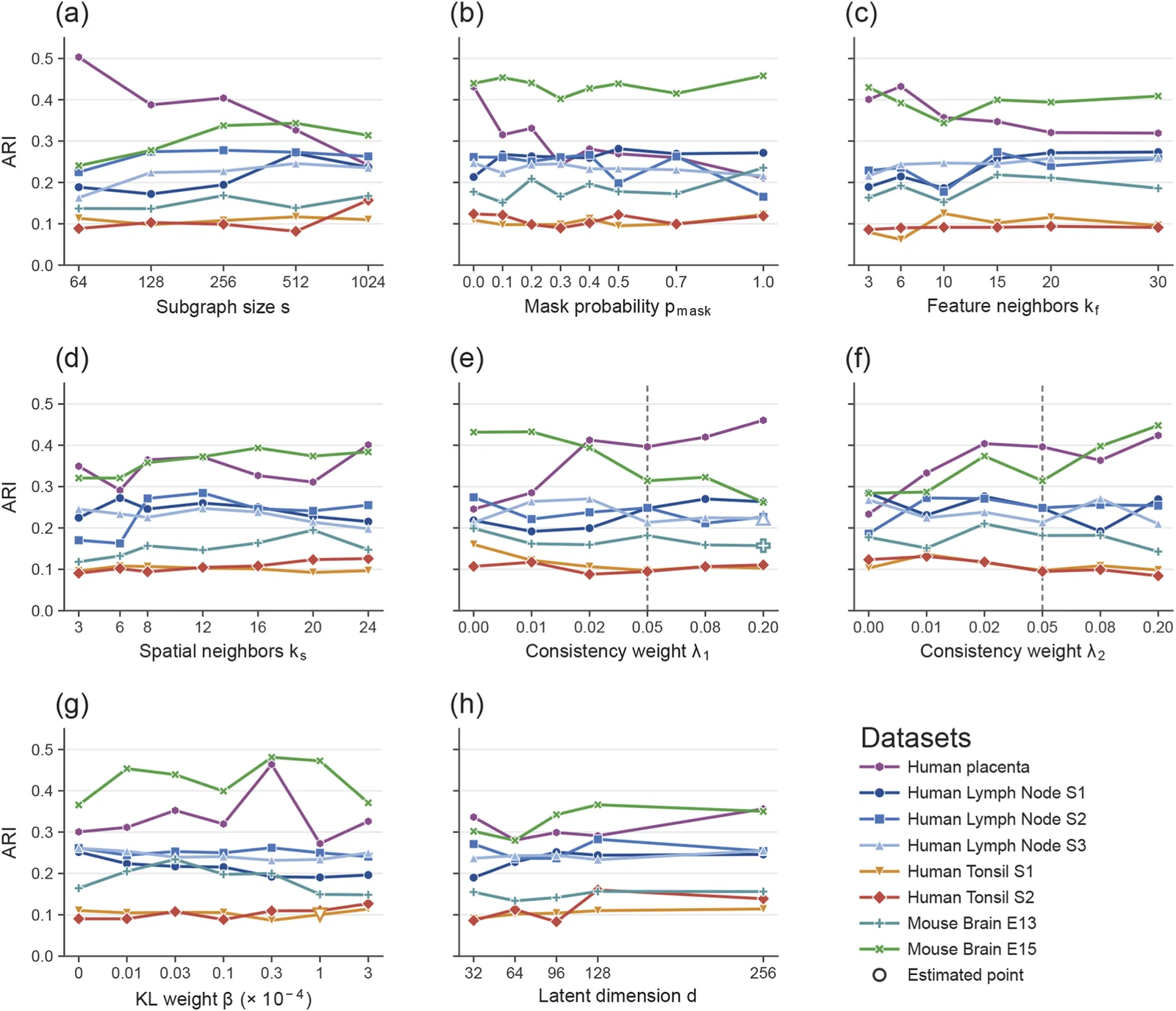
Parameter sensitivity analysis of SubPoE across eight datasets. Panels **(a–h)** show ARI values under different settings of key model parameters.

### 3.5 Uncertainty-aware PoE interpretation

The central premise of PoE fusion is that expert contributions should reflect their reliability at each cell. We tested this premise using the RNA modality of the Human Tonsil S1 dataset. Posterior variances from the molecular-topology and spatially augmented experts were summarized as cell-level uncertainty and projected onto the tissue section (Figures 6a,b).

**FIGURE 6:**
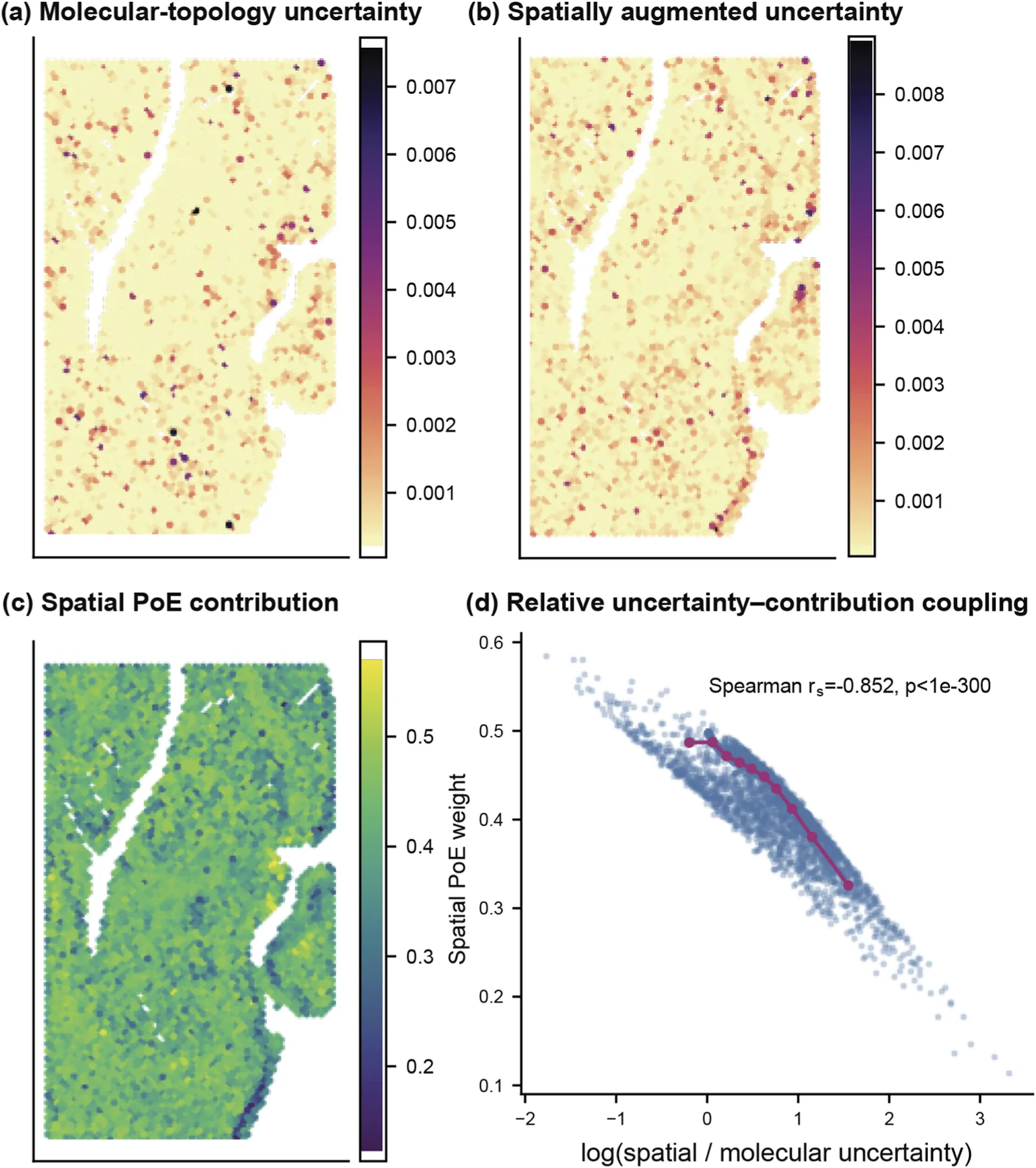
Cell-level interpretation of uncertainty-aware PoE fusion on the RNA modality of the Human Tonsil S1 dataset **(a)** Molecular-topology uncertainty. **(b)** Spatially augmented uncertainty. **(c)** Spatial PoE contribution. **(d)** Relative uncertainty-contribution coupling. The red line indicates the fitted trend, and Spearman’s rank correlation is shown in the panel.

Specifically, the uncertainty of expert *e* for cell *i* was calculated by averaging its posterior variance across the *d* latent dimensions: $u_{i}^{e}=\frac{1}{d}\sum_{k=1}^{d}{\left(\sigma_{ik}^{e}\right)}^{2}$ . Because PoE fusion weights each expert according to its precision, the normalized contribution of the spatial expert was calculated as $w_{i}^{\text{spatia}l}=\frac{{\left(u_{i}^{\text{spatial}}\right)}^{-1}}{{\left(u_{i}^{\text{spatial}}\right)}^{-1}+{\left(u_{i}^{\text{molecular}}\right)}^{-1}}$ . We further quantified the uncertainty of the spatial expert relative to the molecular expert as $r_{i}=\log\frac{u_{i}^{\text{spatial}}}{u_{i}^{\text{molecular}}}$ , and assessed its association with $w_{i}^{\text{spatial}}$ using Spearman’s rank correlation across cells. Additional details on uncertainty aggregation, numerical stabilization, PoE-weight calculation, and correlation analysis are provided in Section 6 of the Supplementary Materials.

Most spatial observations showed low posterior uncertainty in both branches, although localized uncertainty hotspots were present. The spatial-expert contribution also varied across the tissue, indicating observation-specific rather than globally fixed fusion. As expected from the precision-weighted PoE formulation, relative uncertainty was strongly negatively associated with spatial-expert contribution $\left(r_{s}=‐0.852\right),\left(p<10^{‐300}\right)$ . We therefore interpret this correlation as a direct visualization of the PoE decision rule, rather than as independent evidence of its performance advantage.

The practical benefit of PoE fusion was evaluated using the component ablation in Section 3.4. In the w/o PoE variant, the molecular-topology and spatially augmented representations were directly concatenated, thereby removing the precision-based integration of the two expert posteriors while retaining the remaining model components and training settings. Unlike direct concatenation, which preserves both representations without adapting their relative contributions, PoE combines them according to their posterior precision for each spatial observation. Replacing PoE with concatenation consistently reduced all six clustering metrics across the eight datasets (Figure 4), supporting the practical advantage of PoE-based fusion over deterministic concatenation. Thus, the uncertainty-weight relationship in Figure 6 explains how PoE operates, whereas the ablation results provide the corresponding performance evidence.

### 3.6 Robustness of SubPoE to ATAC feature perturbations

To characterize the response of SubPoE to ATAC feature corruption, we selected Mouse Brain E13 as a controlled test case. This dataset contains paired RNA and ATAC measurements, with the high-dimensional and sparse ATAC modality providing a suitable input for evaluating sensitivity to feature corruption. All matched spatial observations and both RNA and ATAC modalities were retained throughout the experiments. Therefore, these experiments evaluated feature-level perturbations within the available ATAC modality rather than the complete absence of a modality. We perturbed the ATAC modality under three conditions.

Gaussian noise was introduced as $X^{\prime }=X+\alpha \epsilon$ , where $\epsilon ∼N\left(0,1\right)$ and α ranged from 0 to 0.5. Feature dropout randomly masked 0%–30% of ATAC entries without removing spatial locations, whereas ATAC signal attenuation scaled the complete ATAC matrix by β , with β decreasing from 1.0 to 0.6. The trained model was evaluated without retraining across five perturbation seeds. For each clustering metric *Q*, repeat-matched performance retention at perturbation level *l* was calculated relative to the unperturbed result obtained with the same seed: $R_{Q}\left(l\right)=\frac{1}{S}\sum_{s=1}^{S}\frac{Q_{l,s}}{Q_{0,s}}$ , where $Q_{l,s}$ and $Q_{0,s}$ denote the perturbed and unperturbed scores for seed *s*, respectively, and *S* = 5. A value of 1 indicates unchanged performance, whereas values below or above 1 indicate decreased or increased performance, respectively. The perturbation-induced change in ATAC-expert uncertainty was calculated as $\Delta u_{\text{ATAC}}\left(l\right)=\frac{1}{S}\sum_{s=1}^{S}\left({\bar{u}}_{\text{ATAC},l,s}-{\bar{u}}_{\text{ATAC},0,s}\right)$ , where ${\bar{u}}_{\text{ATAC}}$ denotes the posterior variance averaged across cells and latent dimensions. Additional details on perturbation generation, feature-dropout sampling, random seeds, and seed-wise result aggregation are provided in Supplementary Section 7 of the Supplementary Materials.

SubPoE remained stable under moderate Gaussian perturbation, with limited changes in ARI, NMI, and FMI up to α=0.2 (Figure 7a). At the strongest noise level α=0.5 , the model retained 71.8% of its baseline ARI, 62.5% of its NMI, and 93.4% of its FMI. Meanwhile, expert uncertainty increased by $4.47\times 10^{-4}$ , showing that posterior variance responded to severe feature corruption. Performance was more stable under feature dropout (Figure 7b). At a 30% dropout rate, NMI retention remained 94.2%, while ARI and FMI retention remained above 1.0. Scaling the ATAC modality to 60% of its original magnitude also produced limited deterioration (Figure 7c). At β=0.6 , SubPoE retained 94.4% of its NMI, while ARI and FMI retention remained above baseline. Overall, SubPoE tolerated moderate perturbations to the available ATAC features, whereas strong Gaussian noise caused a clearer reduction in information-based clustering agreement.

**FIGURE 7:**
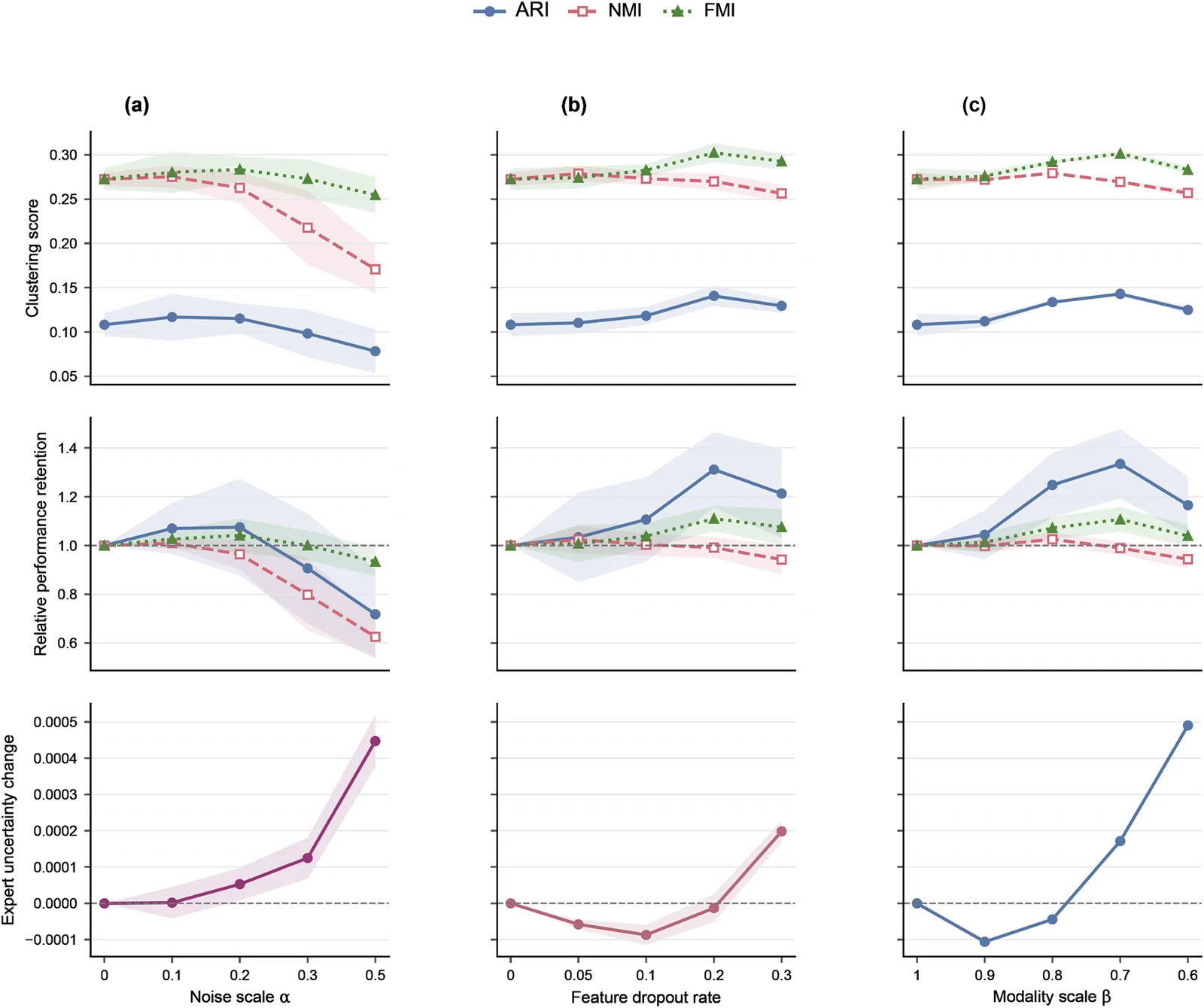
Results on the Mouse Brain E13 dataset under **(a)** Gaussian feature noise, **(b)** feature dropout, and **(c)** signal attenuation. For each condition, the columns show clustering scores, repeat-matched performance retention, and the change in fused ATAC-expert uncertainty. Lines represent means across five perturbation seeds, and shaded regions denote sample standard deviations.

### 3.7 Role and computational efficiency of node-induced subgraph sampling

To determine whether node-induced subgraph sampling serves only to reduce computational cost or also contributes to representation learning, we first evaluated its effect on clustering performance (Figure 8). SubPoE was trained with subgraphs containing 64, 128, 256, 512, or 1,024 nodes on eight datasets, including human placenta, Human Lymph Node S1-S3, Human Tonsil S1-S2, and Mouse Brain E13 and E15. The resulting ARI, NMI, and FMI values were compared with those obtained from full-graph training without subgraph sampling (No SG).

**FIGURE 8:**
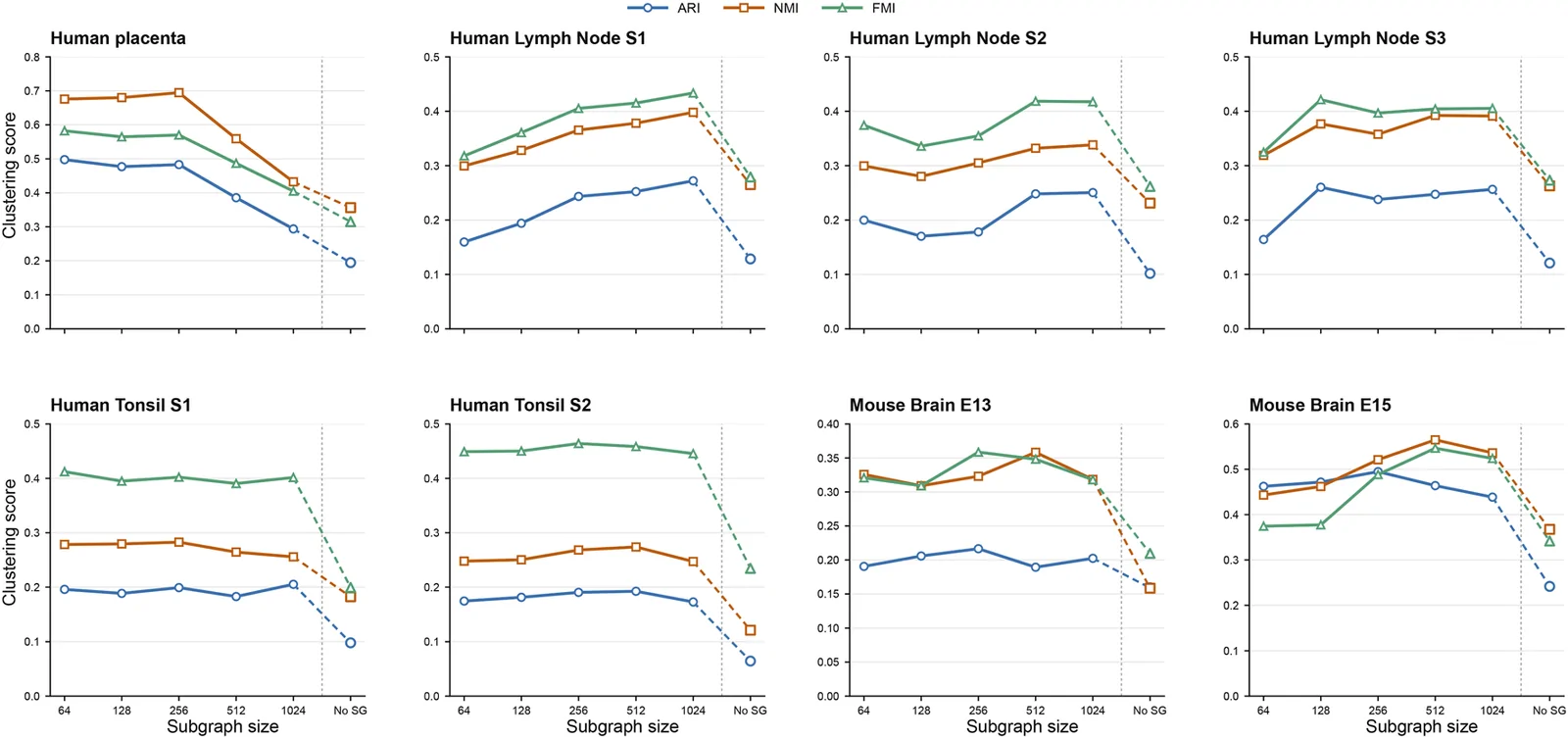
Effect of node-induced subgraph sampling on spatial domain identification. ARI, NMI, and FMI obtained with subgraph sizes of 64, 128, 256, 512, and 1,024 nodes and with full-graph training without subgraph sampling (No SG) across eight spatial multi-omics datasets.

Across the eight datasets, sampled-subgraph training generally achieved higher clustering scores than the No SG setting. For example, on human placenta, the 256-node setting achieved an ARI of 0.483, an NMI of 0.695, and an FMI of 0.570, compared with 0.195, 0.357, and 0.315, respectively, under No SG. On Human Tonsil S2, the 512-node setting achieved corresponding values of 0.192, 0.274, and 0.459, whereas No SG achieved 0.064, 0.121, and 0.235.

Importantly, the No SG model had access to the complete graph but nevertheless showed substantially lower clustering performance. The benefit of subgraph induction therefore cannot be explained solely by reduced computational cost. Instead, the results indicate that subgraph induction is an integral part of representation learning in SubPoE: it controls the spatial and molecular context involved in each optimization step while repeated resampling exposes the model to diverse local neighborhoods. The non-monotonic dependence on subgraph size further suggests a regularization effect, because intermediate subgraphs often performed better than both smaller subgraphs with insufficient context and full-graph training without stochastic resampling. However, the optimal size remained dataset dependent, indicating that subgraph induction balances local-context preservation, stochastic regularization, and graph coverage rather than imposing a universally optimal neighborhood scale.

We next evaluated computational efficiency from two complementary perspectives (Figure 9). Cross-method comparisons on Human Tonsil S1 showed that SubPoE maintained relatively low runtime, peak RAM usage, and VRAM overhead compared with the baseline methods (Figure 9a), demonstrating a competitive computational profile. Complete comparisons of these three metrics across all eight datasets are provided in Supplementary Figures S8–S10, respectively.

**FIGURE 9:**
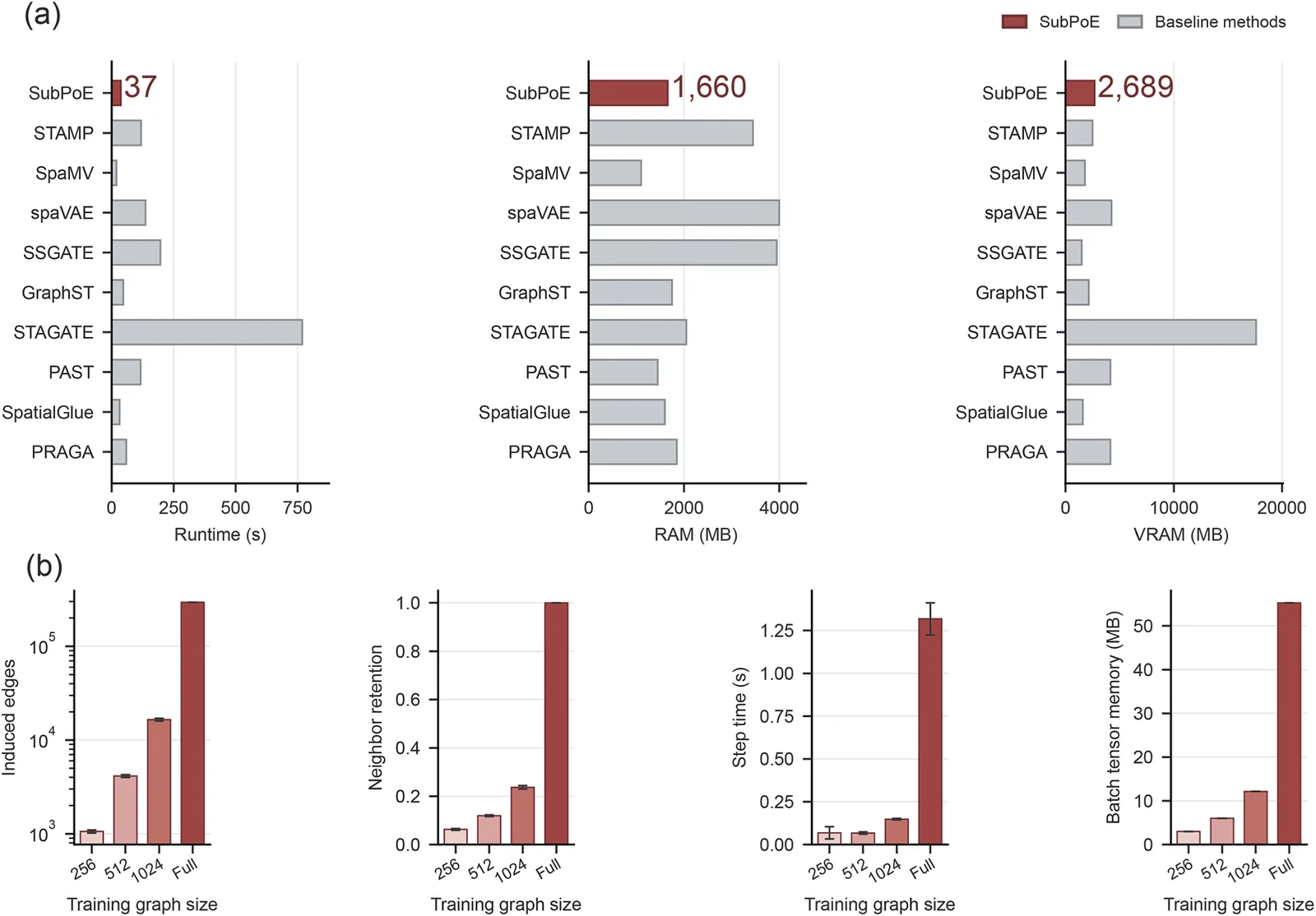
Computational efficiency of SubPoE on Human Tonsil S1. **(a)** Runtime, peak RAM, and VRAM overhead of SubPoE and nine baseline methods; **(b)** Induced edge count, local-neighbor retention, per-step training time, and batch-tensor memory under subgraph sizes of 256, 512, and 1,024 nodes and full-graph training.

We then isolated the efficiency gains attributable to subgraph induction within SubPoE (Figure 9b). For a sampled node set $V_{s}$ , the induced edge set was defined as $\left|E_{s}\right|=\left|\left\{\left(i,j\right)\in E:i,j\in V_{s}\right\}\right|$ . Local-neighbor retention was defined as $R_{\text{nbr}}\left(V_{s}\right)=\frac{1}{\left|V_{s}\right|}\sum_{i\in V_{s}}\frac{\left|N_{i}\cap V_{s}\right|}{\left|N_{i}\right|}$ , where $N_{i}$ denotes the full-graph neighbors of spatial observation *i*. Forward-plus-backward time was measured per optimization step, and batch-tensor memory as the memory occupied by retained tensors. Implementation and profiling details are provided in Section 8 of the Supplementary Materials.

Compared with full-graph training, the 1,024-node setting reduced the induced edge count by approximately 94%, per-step training time by 89%, and batch-tensor memory by 78%, while retaining approximately 24% of local neighbors. Together with the clustering results in Figure 8, these findings show that subgraph induction substantially reduces computational cost while preserving sufficient local structure for effective representation learning.

## 4 Conclusion

In this study, we present SubPoE, a subgraph-induced Product-of-Experts framework for spatial multi-omics integration. Dynamically node-induced subgraphs provide cell-aligned local training views across modality-specific molecular similarity graphs, the shared spatial graph, and multi-omics features. Within each modality, a dual-topology variational graph encoder represents feature and spatial topologies as probabilistic experts, whose evidence is adaptively combined by PoE according to posterior precision. A composite self-supervised objective further constrains the multimodal representation. SubPoE therefore organizes local topology learning, branch-uncertainty modeling and cross-modal self-supervised regularization within a unified framework rather than as a collection of independent modules. Across eight public paired spatial multi-omics datasets, SubPoE achieves competitive and comparatively stable spatial clustering performance against nine representative methods, and the ablation results support the contributions of subgraph induction and PoE fusion.

The current study evaluates paired spatial multi-omics data mainly through spatial clustering-related tasks. Moreover, PoE fusion is presently applied to topology-specific experts within each modality, whereas cross-modal representations are integrated through latent concatenation and self-supervised constraints. Future work could extend SubPoE to broader modality combinations, incomplete-modality settings, and larger spatial datasets, while developing a fully probabilistic cross-modal fusion strategy and incorporating biological priors to improve interpretability. Overall, SubPoE provides a practical framework for integrating complementary molecular and spatial information and offers a flexible foundation for downstream spatial multi-omics analysis.

## Data Availability

The datasets used in this study are publicly available. Human lymph node S1–S3 data are available from GEO (accession GSE263617) (Long et al., 2024). Human tonsil S1–S2 data are available from 10x Genomics (10x Genomics, 2023). Mouse brain E13/E15 data are available from NGDC (accession OEP003285) (Jiang et al., 2023). Human placenta data are available via SCP2601 and Zenodo 10981713 (Ounadjela et al., 2024).
