# Supplementary material for: SubPoE: a subgraph-induced Product-of-Experts framework for spatial multi-omics integration
Source: Front Genet. 2026 Oct 22;17:1977260. doi: 10.3389/fgene.2026.1977260 (PMC13734670; doi:10.3389/fgene.2026.1977260)
Supplement: Supplementary file 1 [file Supplementaryfile1.docx]

***Supplementary Material***

**1. Dataset statistics and preprocessing**

SubPoE was evaluated on eight spatial multi-omics datasets spanning multiple species, tissues, sequencing technologies and modality combinations. The benchmark included three Human Lymph Node sections, two Human Tonsil sections, two developmental Mouse Brain sections and one Human placenta section. Five datasets contained paired RNA and antibody-derived tag (ADT) measurements, and three contained paired RNA and chromatin-accessibility measurements. The datasets were generated using 10x Genomics Visium-based assays [1] and MISAR-seq. This collection provided a heterogeneous benchmark for evaluating SubPoE across immune, developmental and placental tissues with distinct modality combinations and feature dimensions. Table S1 summarizes the datasets included in the evaluation.

Here, $\text{N}_{\text{c}}\text{ }$denotes the number of cells or spatial spots, $\text{N}_{\text{g}\text{ }}$denotes the number of gene features, $\text{N}_{\text{p}\text{ }}$denotes the number of protein features for RNA-ADT datasets, and $\text{N}_{\text{e}}\text{ }$denotes the number of epigenomic features for RNA-ATAC datasets. The values in Table S1 describe the dimensions reported for the datasets before the feature-selection and dimensionality-reduction steps used by SubPoE. For each dataset, molecular matrices, spatial coordinates, and reference annotations were restricted to a common observation set. Reference annotations were used only for downstream evaluation and were not supplied to the representation-learning procedure.

**1.1 Human lymph node datasets**

The Human Lymph Node collection contained three spatial sections, denoted S1, S2 and S3. These datasets were generated using a 10x Genomics Visium-based spatial multi-omics workflow and contained paired RNA and ADT measurements. The three samples comprised 3,359, 3,484 and 3,408 spatial observations, respectively. Each sample contained 18,085 gene features and 31 protein features before preprocessing.

Lymph nodes contain spatially organized immune compartments with closely positioned but functionally distinct cellular populations. The three sections therefore allowed us to assess the reproducibility of spatial-domain identification across related samples. They also provided a setting in which paired transcriptomic and surface-protein signals could be integrated while preserving local tissue organization.

**1.2 Human tonsil datasets**

The Human Tonsil collection consisted of two RNA-ADT samples, referred to as S1 and S2. Both samples were generated using a 10x Genomics Visium-based spatial multi-omics workflow. Human Tonsil S1 and S2 contained 4,519 and 4,521 spatial observations, respectively. Each sample contained 18,085 gene features; S1 contained 31 protein features, whereas S2 contained 35.

These samples represented heterogeneous immune tissues with spatially restricted cellular niches. Their inclusion tested whether SubPoE could recover coherent domains in densely sampled tissue sections while remaining robust to modest differences in protein-panel dimensionality. Human Tonsil S1 was additionally used for the uncertainty-aware PoE interpretation and subgraph-efficiency analyses reported in the main manuscript.

**1.3 MISAR-seq mouse embryonic brain datasets**

The developmental Mouse Brain collection included two MISAR-seq datasets corresponding to embryonic days E13 and E15. MISAR-seq jointly measures gene expression and chromatin accessibility [2] within the same spatial observations. The E13 and E15 datasets contained 1,777 and 1,949 observations, respectively, and each retained 32,285 gene features. Their epigenomic feature dimensions were 123,840 and 141,420, respectively.

These two developmental stages captured distinct spatial and regulatory patterns during embryonic brain development. They provided a demanding RNA-ATAC benchmark because the epigenomic modality was substantially higher-dimensional and sparser than the paired RNA modality. Their inclusion allowed SubPoE to be evaluated on developmental tissue with paired transcriptomic and chromatin-accessibility measurements.

**1.4 Human placenta dataset**

The Human placenta dataset contained paired transcriptomic and epigenomic measurements [3] from 1,662 spatial observations. The source dataset included 36,601 gene features and 63 epigenomic features before preprocessing. Placental tissue contains heterogeneous maternal and fetal compartments and therefore provides a spatial organization distinct from immune and developmental brain tissues.

This dataset differed from the other RNA-ATAC samples in organism, tissue context and relative feature dimensionality. Its inclusion broadened the evaluation beyond mouse embryonic brain and provided an additional test of cross-tissue applicability for spatial transcriptome-epigenome integration.

**1.5 Cross-modal alignment and quality control**

For every paired spatial multi-omics sample, modality-specific matrices were aligned using the intersection of their cell or spot identifiers. Spatial coordinates and reference annotations were reordered using the same identifiers. This procedure established a one-to-one correspondence among molecular measurements, spatial positions, graph nodes and evaluation labels.

Observations without complete paired measurements were removed before graph construction. Features failing the quality-control criteria of the source dataset or preprocessing workflow were also excluded. All filtering and alignment operations were completed before constructing the modality-specific molecular graphs and the shared spatial graph. Consequently, the graphs and feature matrices used within each dataset were defined over the same cells or spots.

**1.6 Modality-specific preprocessing**

RNA counts were processed independently for each dataset. Highly variable genes were selected from the count matrix using the Seurat procedure [4]. The retained counts were normalized for library size, log-transformed and standardized across observations. These steps reduced sequencing-depth effects and the contribution of low-information genes while preserving transcriptomic variation relevant to spatial-domain identification.

ADT and ATAC measurements were transformed using the modality-specific low-dimensional preprocessing implemented in the SubPoE pipeline. The transformation included TF-IDF weighting, row normalization, log stabilization and randomized singular value decomposition [5]. The first latent component was removed where specified by the implementation to reduce dominant technical variation, and the retained components were standardized before graph construction and model training.

Finally, the processed matrices for each modality were independently scaled using column-wise min-max normalization and mapped to the interval [0,1]. This scaling reduced numerical differences among transcriptomic, proteomic and epigenomic inputs. The same processed matrices were used to construct modality-specific feature graphs and to provide node features to the SubPoE encoders.

**2. Evaluation Metrics**

To evaluate spatial-domain identification across the eight spatial multi-omics datasets, we compared the predicted cluster assignments with the corresponding reference annotations using six complementary metrics: normalized mutual information (NMI), adjusted mutual information (AMI) [6], the Fowlkes-Mallows index (FMI), the adjusted Rand index (ARI), V-measure and completeness (Compl). The same metric definitions were applied to SubPoE and all baseline methods. These measures capture complementary aspects of clustering quality, including shared information, pairwise agreement, correction for chance, cluster homogeneity, overlap between co-clustered pairs, and recovery of complete reference domains.

**2.1 Normalized Mutual Information**

NMI rescales MI by the entropies of the two label assignments:

$$\begin{aligned} \text{NMI}\left( \text{C}\text{,}\text{K} \right)\text{=}\frac{\text{MI(}\text{C}\text{,}\text{K}\text{)}}{\sqrt{\text{H}\text{(}\text{C}\text{)}\text{H}\text{(}\text{K}\text{)}}}\#\left( 1 \right) \end{aligned}\text{}$$

where

$$\begin{aligned} \text{H}\left( \text{C} \right)\text{=-}\sum_{\text{i}} \frac{\text{a}_{\text{i}}}{\text{n}}\text{log}\left( \frac{\text{a}_{\text{i}}}{\text{n}} \right)\text{, }\text{H}\left( \text{K} \right)\text{=-}\sum_{\text{j}} \text{ }\frac{\text{b}_{\text{j}}}{\text{n}}\text{log}\left( \frac{\text{b}_{\text{j}}}{\text{n}} \right)\#\left( 2 \right) \end{aligned}\text{}$$

NMI ranges from 0 to 1, with larger values indicating greater agreement between the predicted clusters and reference annotations.

**2.2 Adjusted Mutual Information**

AMI corrects MI for the agreement expected under random label assignments:

$$\begin{aligned} \text{AMI}\left( \text{C}\text{,}\text{K} \right)\text{=}\frac{\text{MI(}\text{C}\text{,}\text{K}\text{)-}\text{E}\text{[MI(}\text{C}\text{,}\text{K}\text{)]}}{\frac{\text{1}}{\text{2}}\left[ \text{H}\text{(}\text{C}\text{)+}\text{H}\text{(}\text{K}\text{)} \right]\text{-}\text{E}\text{[MI(}\text{C}\text{,}\text{K}\text{)]}}\#\left( 3 \right) \end{aligned}\text{}$$

Here,$\text{E}\left[ \text{MI}\left( \text{C}\text{,}\text{K} \right) \right]$denotes the expected mutual information under random clustering with fixed cluster sizes. An AMI close to 1 indicates strong agreement, whereas a value near 0 corresponds to agreement expected by chance.

**2.3 Fowlkes-Mallows Index**

FMI evaluates pairwise agreement [7] between the predicted and reference partitions. Let TP denote the number of observation pairs placed in the same group under both assignments, FP the number placed in the same predicted cluster but different reference classes, and FN the number placed in the same reference class but different predicted clusters. FMI is defined as

$$\begin{aligned} \text{FMI=}\sqrt{\frac{\text{TP}}{\text{TP}\text{+}\text{FP}}\text{⋅}\frac{\text{TP}}{\text{TP}\text{+}\text{FN}}}\#\left( 4 \right) \end{aligned}\text{}$$

FMI ranges from 0 to 1. Higher values indicate that the predicted clusters simultaneously achieve greater pairwise precision and recall.

**2.4 Adjusted Rand Index**

ARI measures pairwise clustering agreement while correcting for chance [8]. Using the contingency-table notation above, ARI is calculated as

$$\begin{aligned} \text{ARI=}\frac{\sum_{\text{ij}} \left( \begin{aligned} \text{n}_{\text{ij}} \\ \text{2} \end{aligned} \right)\text{-}\frac{\sum_{\text{i}} \left( \begin{aligned} \text{a}_{\text{i}} \\ \text{2} \end{aligned} \right)\sum_{\text{j}} \left( \begin{aligned} \text{b}_{\text{j}} \\ \text{2} \end{aligned} \right)}{\left( \begin{aligned} \text{n} \\ \text{2} \end{aligned} \right)}}{\frac{\text{1}}{\text{2}}\left[ \sum_{\text{i}} \left( \begin{aligned} \text{a}_{\text{i}} \\ \text{2} \end{aligned} \right)\text{+}\sum_{\text{j}} \left( \begin{aligned} \text{b}_{\text{j}} \\ \text{2} \end{aligned} \right) \right]\text{-}\frac{\sum_{\text{i}} \left( \begin{aligned} \text{a}_{\text{i}} \\ \text{2} \end{aligned} \right)\sum_{\text{j}} \left( \begin{aligned} \text{b}_{\text{j}} \\ \text{2} \end{aligned} \right)}{\left( \begin{aligned} \text{n} \\ \text{2} \end{aligned} \right)}}\#\left（ 5 \right） \end{aligned}\text{}$$

ARI has a maximum value of 1, indicating identical partitions. Values near 0 indicate chance-level agreement, whereas negative values indicate agreement below that expected by chance.

**2.5 Homogeneity, Completeness, and V-measure**

Homogeneity evaluates whether each predicted cluster contains observations from only one reference class:

$$\begin{aligned} \text{h=1-}\frac{\text{H}\left( \text{C} \mid\text{K} \right)}{\text{H}\left( \text{C} \right)}\#\left（ 6 \right） \end{aligned}\text{}$$

Completeness evaluates whether all observations belonging to the same reference class are assigned to the same predicted cluster:

$$\begin{aligned} \text{c}\text{=1-}\frac{\text{H}\left( \text{K} \mid\text{C} \right)}{\text{H}\left( \text{K} \right)}\#\left（ 7 \right） \end{aligned}\text{}$$

V-measure is the harmonic mean [9] of homogeneity and completeness:

$$\begin{aligned} \text{V}\text{=}\frac{\text{2h}\text{c}}{\text{h+}\text{c}}\#\left（ 8 \right） \end{aligned}\text{}$$

Homogeneity, completeness, and V-measure range from 0 to 1. Higher completeness indicates that each annotated spatial domain is recovered without being fragmented across multiple predicted clusters, whereas a higher V-measure indicates a better balance between cluster purity and domain completeness.

Together, these metrics provide a multidimensional assessment of clustering performance. NMI, and AMI quantify information-level correspondence; FMI and ARI assess pairwise agreement; and V-measure and completeness evaluate the structural consistency of the recovered domains. Because these label-based measures do not directly quantify spatial continuity, they were complemented by qualitative comparisons of spatial clustering maps and low-dimensional embeddings in the main analysis. The metric set follows the manuscript’s stated evaluation protocol and the organizational pattern of the reference supplementary material.

**3. Quantitative evaluation across the benchmark datasets**

To provide the dataset-level evidence underlying the aggregate comparison in the main manuscript, we report the complete quantitative results for SubPoE and nine representative baseline methods on all eight spatial multi-omics datasets. The benchmark comprises Human Lymph Node S1-S3, Human Tonsil S1-S2 with paired RNA-ADT measurements; Mouse Brain E13, E15 and Human placenta with paired RNA-ATAC measurements. The competing methods comprise four spatial transcriptomics approaches (PAST [10], GraphST [11], STAMP [12], and STAGATE [13]) and five spatial multi-omics integration approaches (SpatialGlue [14], SpaMV [15], SSGATE [16], PRAGA [17], and spaVAE [18]). All methods were evaluated on the same aligned observations and reference annotations. Methods restricted to spatial transcriptomics received RNA expression and spatial coordinates, whereas multi-omics methods received the available paired omics profiles together with spatial information.

Tables S2-S9 list NMI, AMI, FMI, ARI, V-measure and completeness for each method on each dataset. These dataset-specific tables complement the aggregate comparison in the main manuscript by reporting all six metrics across the eight datasets. Higher values indicate greater agreement between the predicted partition and the reference spatial domains. The detailed results show that SubPoE maintains a comparatively strong and balanced profile across the metric set. Its advantage is not confined to a single agreement criterion: high values are observed for information-based, pairwise-agreement, overlap-based, and domain-completeness measures. This pattern supports the conclusion that the learned representation preserves class correspondence without improving one aspect of clustering at the expense of another.

Performance nevertheless varies with tissue architecture and modality composition. Individual baseline methods can obtain the best score for a particular dataset or metric, and the numerical differences among leading methods are smaller in some samples than in others. SubPoE is therefore characterized by cross-dataset competitiveness and stability rather than universal dominance in every entry. Its consistently high central tendency across the eight datasets indicates that the combination of cell-aligned subgraph induction, dual-topology encoding, and uncertainty-aware PoE fusion transfers across immune, developmental, and placental tissues as well as across RNA-ADT and RNA-ATAC assays. The complete tables also make the method-specific and dataset-specific variation visible beyond the aggregate distributions reported in the main text.

**4. Qualitative evaluation on the supplementary datasets**

The main manuscript presents spatial cluster maps and UMAP embeddings for Human Lymph Node S1, a representative RNA-ADT immune-tissue dataset. To broaden the qualitative assessment, Figs. S1-S7 provide the corresponding results for the remaining seven datasets: Human Lymph Node S2 and S3, Human Tonsil S1 and S2, Mouse Brain E13 and E15, and Human placenta. Each figure follows the same model order, color convention and visualization procedure as the main manuscript, enabling direct comparison among reference annotations, original modalities, SubPoE and baseline methods.

Across the supplementary RNA-ADT immune-tissue datasets, SubPoE generally produced spatial domains that followed the overall tissue geometry while limiting isolated assignments and excessive fragmentation. The associated UMAP embeddings showed comparatively compact groups and reduced mixing between neighboring clusters. These results indicate that combining molecular-similarity and spatial-neighborhood evidence helps recover local domains in lymph node and tonsil tissues.

The Mouse Brain E13, Mouse Brain E15 and Human placenta datasets further assessed SubPoE under paired RNA-ATAC measurements. In these samples, SubPoE recovered spatially coherent regions compatible with the large-scale reference organization and generated low-dimensional embeddings with clearer regional structure than either modality alone in many areas. Taken together, the seven supplementary cases support the applicability of SubPoE across related immune-tissue sections and RNA-ATAC spatial multi-omics datasets.

**5. Computational Efficiency and Complexity**

To evaluate the computational cost of SubPoE, we compared its end-to-end training time, system memory usage and GPU memory usage with nine baseline methods across eight spatial multi-omics datasets (Figs. S8-S10). Overall, SubPoE showed moderate training time and memory consumption across datasets. Although several lightweight baselines were faster or required less memory in some settings, SubPoE consistently avoided the high computational overhead observed for more resource-intensive graph-based methods, particularly STAGATE, SSGATE and spaVAE. Its additional dual-topology encoding and uncertainty-aware PoE fusion therefore did not introduce prohibitive resource demands. Taken together, these results indicate that SubPoE achieves a practical balance between representation-learning performance and computational efficiency across tissues and modality combinations.

**6. Uncertainty aggregation, PoE-weight calculation and correlation analysis**

SubPoE represents the molecular-topology expert and the spatially augmented expert within each modality as diagonal Gaussian posteriors. For cell *i*, expert $\text{e}\text{∈}\left\{ \text{m} , \text{s} \right\}$ outputs a latent mean $\text{μ}_{\text{i}\text{,}\text{e}}\text{∈}\text{R}^{\text{d}}$ and posterior variance $\text{σ}_{\text{i}\text{,}\text{e}}^{\text{2}}\text{∈}\text{R}^{\text{d}}$, where *m* and *s* denote the molecular-topology and spatially augmented experts, respectively. To obtain a cell-level uncertainty score for visualization and correlation analysis, we averaged the posterior variance across latent dimensions:

$$\text{u}_{\text{i}\text{,}\text{e}}\text{=}\frac{\text{1}}{\text{d}}\sum_{\text{l}\text{=1}}^{\text{d}} \text{σ}_{\text{i}\text{,}\text{e}\text{,}\text{l}}^{\text{2}}\text{}$$

This scalar summarizes the overall uncertainty of each expert for a given cell while preserving cell-level correspondence between the two topology branches.

For PoE fusion, each expert contributes according to its posterior precision. The dimension-wise precision of expert *e* was defined as

$$\text{τ}_{\text{i}\text{,}\text{e}\text{,}\text{l}}\text{=}\frac{\text{1}}{\text{σ}_{\text{i}\text{,}\text{e}\text{,}\text{l}}^{\text{2}}\text{+ϵ }}\text{}$$

where $\text{ϵ}$ is a small positive constant used to avoid division by zero. The fused posterior was then computed as

$$\text{σ}_{\text{i}\text{,PoE,}\text{l}}^{\text{2}}\text{=}\left( \sum_{\text{e}} \text{τ}_{\text{i}\text{,}\text{e}\text{,}\text{l}} \right)^{\text{-1}}\text{,}\text{}$$

$$\text{μ}_{\text{i}\text{,PoE,}\text{l}}\text{=}\text{σ}_{\text{i}\text{,PoE,}\text{l}}^{\text{2}}\sum_{\text{e}} \text{τ}_{\text{i}\text{,}\text{e}\text{,}\text{l}}\text{μ}_{\text{i}\text{,}\text{e}\text{,}\text{l}}\text{.}\text{}$$

Thus, experts with lower posterior variance have higher precision and contribute more strongly to the fused latent representation. For the cell-level interpretation in Fig. 6, the normalized contribution of the spatial expert was calculated from the aggregated uncertainty:

$$\text{ω}_{\text{i}\text{,}\text{s}}\text{=}\frac{\left( \text{u}_{\text{i}\text{,}\text{s}}\text{+ϵ} \right)^{\text{-1}}}{\left( \text{u}_{\text{i}\text{,}\text{m}}\text{+ϵ} \right)^{\text{-1}}\text{+}\left( \text{u}_{\text{i}\text{,}\text{s}}\text{+ϵ} \right)^{\text{-1}}}\text{.}\text{}$$

The molecular expert contribution is $\text{1-}\text{ω}_{\text{i}\text{,}\text{s}}$.

To quantify whether PoE weighting followed the relative reliability of the two experts, we defined the relative spatial uncertainty as

$$\text{r}_{\text{i}}\text{=log}\frac{\text{u}_{\text{i}\text{,}\text{s}}\text{+ϵ}}{\text{u}_{\text{i}\text{,}\text{m}}\text{+ϵ}}\text{.}\text{}$$

A positive value indicates that the spatial expert is more uncertain than the molecular-topology expert, whereas a negative value indicates lower spatial uncertainty. We then computed Spearman’s rank correlation between $\text{r}_{\text{i}}$ and $\text{ω}_{\text{i}\text{,}\text{s}}$ across cells. This rank-based analysis was used because it assesses monotonic association without assuming a linear relationship between uncertainty and contribution. A negative correlation indicates that cells with higher relative spatial uncertainty receive lower spatial-expert weights, consistent with the reliability-adaptive behavior expected from PoE fusion.

Numerical stabilization was applied consistently when converting posterior variance into precision or log-ratio quantities. Specifically, $\text{ϵ}$ was added to all variance-based denominators and logarithmic ratios. This prevents unstable values for cells with very small posterior variance and ensures that uncertainty maps, PoE weights and correlation statistics are computed from finite quantities.

**7. Perturbation generation and seed-wise aggregation**

To evaluate the robustness of SubPoE to ATAC feature corruption, perturbations were applied to the ATAC modality of the Mouse Brain E13 dataset after model training. The trained model parameters were kept fixed, and the perturbed ATAC inputs were evaluated without retraining. This design isolates the response of the learned representation and uncertainty estimates to controlled input corruption, rather than measuring variability caused by re-optimization.

Let $\text{X}_{\text{ATAC}}\text{∈}\text{R}^{\text{n}\text{×}\text{p}}$ denote the original ATAC feature matrix. For Gaussian feature noise, the perturbed matrix was generated as

$${\text{X}^{\text{'}}}_{\text{ATAC}}\text{=}\text{X}_{\text{ATAC}}\text{+}\text{α}\text{ϵ, ϵ\textasciitilde}\text{N}\left( \text{0} , \text{1} \right)\text{,}\text{}$$

where $\text{α}$ controls the noise strength and ranged from 0 to 0.5.

For feature dropout, a binary mask $\text{M}_{\text{ρ}\text{,}\text{s}}\text{∈}\left\{ \text{0} , \text{1} \right\}^{\text{n}\text{×}\text{p}}$ was sampled under dropout rate $\text{ρ}$, and the perturbed matrix was computed as

$${\text{X}^{\text{'}}}_{\text{ATAC}}\text{=}\text{X}_{\text{ATAC}}\odot\text{M}_{\text{ρ}\text{,}\text{s}}\text{.}\text{}$$

The dropout rate ranged from 0 to 30%. Selected ATAC entries were set to zero, while all cells and spatial coordinates were retained. Thus, feature dropout introduced controlled entry-level signal loss without changing tissue geometry or observation-level correspondence. For signal attenuation, the ATAC matrix was scaled as

$${\text{X}^{\text{'}}}_{\text{ATAC}}\text{=}\text{β}\text{X}_{\text{ATAC}}\text{,}\text{}$$

where $\text{β}$ decreased from 1.0 to 0.6, simulating a global attenuation of ATAC signal intensity.

For each perturbation condition and perturbation level, five independent perturbation seeds were used. The seed controlled the random sampling of Gaussian noise or feature-dropout masks. The same trained model was used across all perturbation seeds, and the unperturbed evaluation corresponding to each seed was used as the matched baseline. This repeat-matched design reduces the effect of seed-specific variation when estimating performance retention.

For each clustering metric *Q*, including ARI, NMI and FMI, seed-wise retention at perturbation level *l* was defined as

$$\text{R}_{\text{Q}\text{,}\text{l}\text{,}\text{s}}\text{=}\frac{\text{Q}_{\text{l}\text{,}\text{s}}}{\text{Q}_{\text{0,}\text{s}}}\text{,}\text{}$$

where $\text{Q}_{\text{l}\text{,}\text{s}}$ is the score under perturbation level *l* and seed *s*, and $\text{Q}_{\text{0,}\text{s}}$ is the corresponding unperturbed score under the same seed index. The reported retention was averaged across the *S*=5 seeds:

$$\text{R}_{\text{Q}}\left( \text{l} \right)\text{=}\frac{\text{1}}{\text{S}}\sum_{\text{s}\text{=1}}^{\text{S}} \text{R}_{\text{Q}\text{,}\text{l}\text{,}\text{s}}\text{.}$$

A retention value of 1 indicates unchanged performance relative to the matched unperturbed baseline, whereas values below or above 1 indicate decreased or increased performance, respectively. For visualization, lines represent seed-wise means and shaded regions denote sample standard deviations.

The perturbation-induced change in ATAC-expert uncertainty was also calculated in a seed-wise manner. For each perturbation level *l* and seed *s*, we first computed the mean ATAC-expert uncertainty, denoted as ${\bar{\text{u}}}_{\text{ATAC,}\text{l}\text{,}\text{s}}$, by averaging the posterior variance across cells and latent dimensions. The change in ATAC-expert uncertainty was then calculated relative to the matched unperturbed baseline:

$$\text{Δ}\text{u}_{\text{ATAC}}\left( \text{l} \right)\text{=}\frac{\text{1}}{\text{S}}\sum_{\text{s}\text{=1}}^{\text{S}} \left( {\bar{\text{u}}}_{\text{ATAC,}\text{l}\text{,}\text{s}}\text{-}{\bar{\text{u}}}_{\text{ATAC,0,}\text{s}} \right)\text{,}\text{}$$

where *S*=5 denotes the number of perturbation seeds. Positive values indicate increased posterior uncertainty after perturbation, whereas values close to zero indicate limited uncertainty change. This seed-wise aggregation links changes in clustering performance with the uncertainty response of the ATAC branch under controlled feature corruption.

**8. Implementation and profiling of subgraph efficiency**

To evaluate the computational efficiency of node-induced subgraph training, we profiled SubPoE on the Human Tonsil S1 dataset using different training graph sizes. The profiled settings included induced subgraphs with 256, 512 and 1,024 nodes, together with the full-graph setting. For each subgraph setting, a node subset was first sampled from the full cell set. The same subset was then used to slice the multi-omics feature matrices and induce the corresponding molecular and spatial graph structures, thereby preserving cell-level correspondence between features and topologies during profiling.

For a sampled node set *B*, the induced edge count was defined as the number of full-graph edges whose two endpoints were both retained in *B* . Local-neighbor retention was used to quantify how much of the original local topology was preserved after subgraph induction. It was calculated as the average proportion of full-graph neighbors retained for cells in the sampled node set:

$$\text{Retention}\left( \text{B} \right)\text{=}\frac{\text{1}}{\left| \text{B} \right|}\sum_{\text{i}\text{∈}\text{B}} \frac{\left| \text{N}\left( \text{i} \right)\text{∩}\text{B} \right|}{\left| \text{N}\left( \text{i} \right) \right|}\text{,}\text{}$$

where $\text{N}\left( \text{i} \right)$ denotes the neighbor set of cell *i* in the full graph. The full-graph setting therefore has a retention value of 1.

Forward-plus-backward time was measured as the wall-clock time required for one optimization step. Each step included the forward pass, loss calculation and backward gradient computation for the current training graph. Data preprocessing, graph construction, model initialization and downstream clustering evaluation were excluded from this timing measurement. This definition focuses on the per-step computational cost directly affected by the number of nodes and induced edges processed during training.

Batch-tensor memory was defined as the memory occupied by retained tensors in the current training step. These tensors included the subgraph feature tensors, graph-index tensors and topology-specific tensors used by the model during forward and backward computation. This measurement was intended to capture the memory cost that changes with training graph size, rather than total system memory or total GPU memory. Therefore, model parameters, optimizer states and unrelated runtime overhead were not included in the batch-tensor memory estimate.

All profiling experiments used the same model architecture and training configuration, with only the training graph size varied. This controlled setup allowed the changes in induced edge count, local-neighbor retention, forward-plus-backward time and batch-tensor memory to be attributed to node-induced subgraph sampling. The profiling results therefore complement the clustering-performance comparison by showing how smaller induced subgraphs reduce per-step computational cost while retaining part of the local graph structure.

**References**

[1] Pappalardo A, Batra K, Perez-Lorenzo R, et al. 10 X Visium Spatial Gene Expression - Fixed Frozen Tissue Processing with CytAssist v1. 2024.

[2] Jiang F, Zhou X, Qian Y, et al. Simultaneous profiling of spatial gene expression and chromatin accessibility during mouse brain development. Nat Methods. 2023 Jul;20(7):1048-1057.

[3] Ounadjela JR, Zhang K, Kobayashi-Kirschvink KJ, et al. Spatial multiomic landscape of the human placenta at molecular resolution. Nat Med. 2024 Dec;30(12):3495-3508.

[4] Stuart T, Butler A, Hoffman P, et al. Comprehensive Integration of Single-Cell Data. Cell. 2019 Jun 13;177(7):1888-1902.e21.

[5] Stuart T, Srivastava A, Madad S, et al. Single-cell chromatin state analysis with Signac. Nat Methods. 2021 Nov;18(11):1333-1341.

[6] Nguyen X V, Epps J, Bailey J. Information theoretic measures for clusterings comparison: is a correction for chance necessary?. Journal of Machine Learning Research, 2010, 11(1):2837-2854.

[7] E,B,Fowlkes, et al. A Method for Comparing Two Hierarchical Clusterings. Journal of the American Statistical Association, 1983.

[8] Hubert L, Arabie P. Comparing partitions. J. Classif, 1985, 2(1):193-218.

[9] Rosenberg A, Hirschberg J. V-Measure: A conditional entropy-based external cluster evaluation. DBLP, 2007.

[10] Li Z, Chen X, Zhang X, et al. Latent feature extraction with a prior-based self-attention framework for spatial transcriptomics. Genome Research, 2023, 33(10):18.

[11] Long Y, Ang KS, Li M, et al. Spatially informed clustering, integration, and deconvolution of spatial transcriptomics with GraphST. Nat Commun. 2023 Mar 1;14(1):1155.

[12] Zhong C, Ang KS, Chen J. Interpretable spatially aware dimension reduction of spatial transcriptomics with STAMP. Nat Methods. 2024 Nov;21(11):2072-2083.

[13] Dong K, Zhang S. Deciphering spatial domains from spatially resolved transcriptomics with an adaptive graph attention auto-encoder. Nat Commun. 2022 Apr 1;13(1):1739.

[14] Long Y, Ang KS, Sethi R, et al. Deciphering spatial domains from spatial multi-omics with SpatialGlue. Nat Methods. 2024 Sep;21(9):1658-1667.

[15] Liu Y, Ma K, Xu H, et al. Interpretable spatial multi-omics data integration and dimensionality reduction with SpaMV. Nat Commun. 2026 Jul 1;17(1):8213.

[16] Lv T, Zhang Y, Liu J, et al. Multi-omics integration for both single-cell and spatially resolved data based on dual-path graph attention auto-encoder. Brief Bioinform. 2024 Jul 25;25(5):bbae450.

[17] Huang X, Ma Z, Meng D, et al. PRAGA: Prototype-aware Graph Adaptive Aggregation for Spatial Multi-modal Omics Analysis. 2024.

[18] Tian T, Zhang J, Lin X, et al, Hakonarson H. Dependency-aware deep generative models for multitasking analysis of spatial omics data. Nat Methods. 2024 Aug;21(8):1501-1513.

**Supplementary Figures and Tables**

**Table S1. Summary of the spatial multi-omics datasets used in this study.**

| **Data source** | **Type** | **Tissue** | **Nc** | **Ng** | **(Np/Ne)** |
| --- | --- | --- | --- | --- | --- |
| 10x Genomics Visium | RNA+ADT | Human_Lymph _Node_ S1 | 3359 | 18085 | 31 |
|  |  | Human_Lymph _Node_S2 | 3484 | 18085 | 31 |
|  |  | Human_Lymph _Node_S3 | 3408 | 18085 | 31 |
|  |  | Human_Tonsil _S1 | 4519 | 18085 | 31 |
|  |  | Human_Tonsil _S2 | 4521 | 18085 | 35 |
| MISAR-seq | RNA+ATAC | Mouse_Brain _E13 | 1777 | 32285 | 123840 |
|  |  | Mouse_Brain _E15 | 1949 | 32285 | 141420 |
| 10x Genomics Visium |  | Human placenta | 1662 | 36601 | 63 |

**Table S2. Quantitative Experimental Evaluation of Multiple Models on Human Lymph Node S1.**

| Methods | NMI(%) | AMI(%) | FMI(%) | ARI(%) | V-measure(%) | Compl.(%) |
| --- | --- | --- | --- | --- | --- | --- |
| **Human Lymph Node S1** | | | | | | |
| PRAGA | 37.85 | 37.44 | 41.17 | 26.60 | 37.85 | 34.66 |
| SpatialGlue | 38.35 | 37.93 | 41.86 | 26.72 | 38.35 | 35.90 |
| PAST | 35.47 | 35.03 | 36.78 | 20.26 | 35.47 | 32.99 |
| STAGATE | 17.76 | 17.25 | 27.05 | 12.12 | 17.76 | 15.58 |
| GraphST | 38.84 | 38.41 | 42.36 | 24.15 | 38.84 | 38.47 |
| SSGATE | 9.77 | 8.47 | 12.39 | 1.90 | 9.77 | 7.47 |
| spaVAE | 35.12 | 34.71 | 33.97 | 19.80 | 35.12 | 30.96 |
| SpaMV | 36.90 | 36.49 | 38.32 | 24.10 | 36.90 | 33.09 |
| STAMP | 36.26 | 35.86 | 33.35 | 18.92 | 36.26 | 32.04 |
| SubPoE | 38.91 | 38.49 | 43.40 | 27.32 | 38.91 | 37.68 |

**Table S3. Quantitative Experimental Evaluation of Multiple Models on Human Lymph Node S2.**

| Methods | NMI(%) | AMI(%) | FMI(%) | ARI(%) | V-measure(%) | Compl.(%) |
| --- | --- | --- | --- | --- | --- | --- |
| **Human Lymph Node S2** | | | | | | |
| PRAGA | 33.53 | 33.04 | 35.30 | 19.28 | 33.53 | 29.93 |
| SpatialGlue | 33.65 | 33.15 | 39.68 | 24.12 | 33.65 | 30.18 |
| PAST | 32.45 | 31.92 | 41.59 | 23.32 | 32.45 | 31.10 |
| STAGATE | 9.07 | 8.41 | 19.27 | 2.78 | 9.07 | 7.90 |
| GraphST | 33.91 | 33.39 | 39.62 | 22.46 | 33.91 | 31.55 |
| SSGATE | 7.53 | 5.89 | 11.57 | 1.18 | 7.53 | 5.68 |
| spaVAE | 22.89 | 22.33 | 27.09 | 10.88 | 22.89 | 20.07 |
| SpaMV | 33.26 | 32.76 | 35.85 | 20.11 | 33.26 | 29.50 |
| STAMP | 29.76 | 29.24 | 31.11 | 15.22 | 29.76 | 26.14 |
| SubPoE | 33.57 | 33.00 | 41.81 | 25.87 | 33.57 | 32.14 |

**Table S4. Quantitative Experimental Evaluation of Multiple Models on Human Lymph Node S3.**

| Methods | NMI(%) | AMI(%) | FMI(%) | ARI(%) | V-measure(%) | Compl.(%) |
| --- | --- | --- | --- | --- | --- | --- |
| **Human Lymph Node S3** | | | | | | |
| PRAGA | 39.71 | 39.29 | 42.57 | 27.38 | 39.71 | 37.49 |
| SpatialGlue | 36.75 | 36.33 | 38.44 | 23.14 | 36.75 | 33.96 |
| PAST | 33.08 | 32.63 | 33.85 | 18.43 | 33.08 | 30.20 |
| STAGATE | 10.78 | 10.22 | 20.33 | 5.21 | 10.78 | 9.44 |
| GraphST | 40.20 | 39.77 | 46.11 | 27.81 | 40.20 | 40.87 |
| SSGATE | 4.72 | 3.38 | 11.35 | 0.64 | 4.72 | 3.66 |
| spaVAE | 25.83 | 25.36 | 26.97 | 12.10 | 25.83 | 22.66 |
| SpaMV | 37.28 | 36.87 | 36.39 | 21.70 | 37.28 | 33.67 |
| STAMP | 33.57 | 33.14 | 33.34 | 18.32 | 33.57 | 30.22 |
| SubPoE | 38.57 | 38.15 | 40.24 | 25.02 | 38.57 | 36.36 |

**Table S5. Quantitative Experimental Evaluation of Multiple Models on Human placenta.**

| Methods | NMI(%) | AMI(%) | FMI(%) | ARI(%) | V-measure(%) | Compl.(%) |
| --- | --- | --- | --- | --- | --- | --- |
| **Human placenta** | | | | | | |
| PRAGA | 12.36 | 10.63 | 15.28 | 3.62 | 12.36 | 10.54 |
| SpatialGlue | 22.37 | 20.82 | 23.23 | 10.86 | 22.37 | 19.47 |
| PAST | 56.28 | 55.40 | 49.68 | 39.50 | 56.28 | 48.84 |
| STAGATE | 12.75 | 11.01 | 16.87 | 4.13 | 12.75 | 11.15 |
| GraphST | 66.59 | 65.93 | 50.10 | 39.23 | 66.59 | 56.92 |
| SSGATE | 45.34 | 44.33 | 36.66 | 25.43 | 45.34 | 39.12 |
| spaVAE | 5.01 | 3.18 | 12.26 | 1.41 | 5.01 | 4.21 |
| SpaMV | 31.44 | 30.05 | 28.93 | 17.02 | 31.44 | 27.38 |
| STAMP | 67.27 | 66.63 | 54.85 | 44.10 | 67.27 | 57.47 |
| SubPoE | 69.98 | 69.59 | 63.22 | 55.58 | 69.98 | 65.81 |

**Table S6. Quantitative Experimental Evaluation of Multiple Models on Human Tonsil S1.**

| Methods | NMI(%) | AMI(%) | FMI(%) | ARI(%) | V-measure(%) | Compl.(%) |
| --- | --- | --- | --- | --- | --- | --- |
| **Human Tonsil S1** | | | | | | |
| PRAGA | 30.92 | 30.85 | 49.35 | 18.10 | 30.92 | 29.05 |
| SpatialGlue | 19.16 | 19.09 | 39.84 | 7.81 | 19.16 | 17.25 |
| PAST | 22.51 | 22.44 | 44.21 | 11.01 | 22.51 | 21.15 |
| STAGATE | 1.78 | 1.70 | 33.33 | 0.05 | 1.78 | 1.59 |
| GraphST | 24.75 | 24.68 | 44.64 | 14.04 | 24.75 | 22.61 |
| SSGATE | 1.21 | 1.03 | 24.30 | 0.09 | 1.21 | 0.92 |
| spaVAE | 1.00 | 0.92 | 32.92 | 0.57 | 1.00 | 0.88 |
| SpaMV | 26.02 | 25.95 | 45.28 | 14.67 | 26.02 | 23.87 |
| STAMP | 22.54 | 22.47 | 41.64 | 12.61 | 22.54 | 19.94 |
| SubPoE | 27.49 | 27.42 | 47.02 | 19.26 | 27.49 | 24.61 |

**Table S7. Quantitative Experimental Evaluation of Multiple Models on Human Tonsil S2.**

| Methods | NMI(%) | AMI(%) | FMI(%) | ARI(%) | V-measure(%) | Compl.(%) |
| --- | --- | --- | --- | --- | --- | --- |
| **Human Tonsil S2** | | | | | | |
| PRAGA | 27.26 | 27.20 | 49.46 | 19.78 | 27.26 | 25.89 |
| SpatialGlue | 24.65 | 24.59 | 47.10 | 18.38 | 24.65 | 22.94 |
| PAST | 16.21 | 16.14 | 44.92 | 14.19 | 16.21 | 15.25 |
| STAGATE | 1.01 | 0.93 | 32.32 | 0.21 | 1.01 | 0.90 |
| GraphST | 21.43 | 21.36 | 45.30 | 16.76 | 21.43 | 19.72 |
| SSGATE | 0.90 | 0.71 | 24.14 | 0.84 | 0.90 | 0.68 |
| spaVAE | 1.17 | 1.09 | 32.04 | 0.41 | 1.17 | 1.05 |
| SpaMV | 18.83 | 18.76 | 42.56 | 12.52 | 18.83 | 17.40 |
| STAMP | 25.66 | 25.59 | 48.64 | 19.35 | 25.66 | 24.42 |
| SubPoE | 28.61 | 28.52 | 45.53 | 19.56 | 28.61 | 24.93 |

**Table S8. Quantitative Experimental Evaluation of Multiple Models on Mouse Brain E13 MISAR.**

| Methods | NMI(%) | AMI(%) | FMI(%) | ARI(%) | V-measure(%) | Compl.(%) |
| --- | --- | --- | --- | --- | --- | --- |
| **Mouse Brain E13 MISAR** | | | | | | |
| PRAGA | 28.67 | 27.61 | 31.39 | 13.01 | 28.67 | 26.13 |
| SpatialGlue | 28.87 | 27.85 | 27.41 | 11.19 | 28.87 | 25.29 |
| PAST | 35.01 | 34.08 | 34.60 | 19.03 | 35.01 | 31.01 |
| STAGATE | 7.94 | 6.63 | 21.11 | 4.95 | 7.94 | 6.87 |
| GraphST | 20.68 | 19.51 | 26.49 | 5.70 | 20.68 | 19.27 |
| SSGATE | 4.16 | 2.79 | 18.57 | 0.20 | 4.16 | 3.72 |
| spaVAE | 14.34 | 13.10 | 23.36 | 5.97 | 14.34 | 12.69 |
| SpaMV | 41.46 | 40.60 | 42.83 | 27.45 | 41.46 | 37.56 |
| STAMP | 32.01 | 31.04 | 32.70 | 17.21 | 32.01 | 27.95 |
| SubPoE | 34.37 | 33.26 | 37.59 | 22.16 | 34.37 | 30.19 |

**Table S9. Quantitative Experimental Evaluation of Multiple Models on Mouse Brain E15 MISAR.**

| Methods | NMI(%) | AMI(%) | FMI(%) | ARI(%) | V-measure(%) | Compl.(%) |
| --- | --- | --- | --- | --- | --- | --- |
| **Mouse Brain E15 MISAR** | | | | | | |
| PRAGA | 53.27 | 52.54 | 53.17 | 43.71 | 53.27 | 53.03 |
| SpatialGlue | 59.47 | 58.87 | 54.03 | 45.68 | 59.47 | 56.99 |
| PAST | 55.48 | 54.83 | 49.63 | 40.67 | 55.48 | 52.61 |
| STAGATE | 9.20 | 7.88 | 21.37 | 8.53 | 9.20 | 8.68 |
| GraphST | 60.93 | 60.34 | 59.05 | 51.35 | 60.93 | 59.51 |
| SSGATE | 8.70 | 7.69 | 18.41 | 4.45 | 8.70 | 8.56 |
| spaVAE | 14.07 | 12.82 | 19.19 | 6.68 | 14.07 | 13.20 |
| SpaMV | 50.22 | 49.48 | 38.23 | 27.20 | 50.22 | 48.36 |
| STAMP | 54.12 | 53.46 | 47.36 | 38.12 | 54.12 | 50.49 |
| SubPoE | 57.57 | 56.93 | 57.22 | 49.38 | 57.57 | 55.37 |

**Fig. S1. Spatial clustering patterns and UMAP visualizations generated by different models on the Human Lymph Node S2 dataset.**

**Fig. S2. Spatial clustering patterns and UMAP visualizations generated by different models on the Human Lymph Node S3 dataset.**

**Fig. S3. Spatial clustering patterns and UMAP visualizations generated by different models on the Human Tonsil S1 dataset.**

**Fig. S4. Spatial clustering patterns and UMAP visualizations generated by different models on the Human Tonsil S2 dataset.**

**Fig. S5. Spatial clustering patterns and UMAP visualizations generated by different models on the Mouse Brain E13 dataset.**

**Fig. S6. Spatial clustering patterns and UMAP visualizations generated by different models on the Mouse Brain E15 dataset.**

**Fig. S7. Spatial clustering patterns and UMAP visualizations generated by different models on the Human placenta dataset.**

**Fig. S8. Comparison of Training Time consumption between SubPoE and nine baseline methods across eight spatial multi-omics datasets.**

**Fig. S9. Comparison of RAM consumption between SubPoE and nine baseline methods across eight spatial multi-omics datasets.**

**Fig. S10. Comparison of VRAM consumption between SubPoE and nine baseline methods across eight spatial multi-omics datasets.**
